# Distinguishing enzymes using metabolome data for the hybrid dynamic/static method

**DOI:** 10.1186/1742-4682-4-19

**Published:** 2007-05-20

**Authors:** Nobuyoshi Ishii, Yoichi Nakayama, Masaru Tomita

**Affiliations:** 1 Institute for Advanced Biosciences, Keio University, Tsuruoka, 997-0035, Japan; 2 Network Biology Research Centre, Articell Systems Corporation, Keio Fujisawa Innovation Village, 4489 Endo, Fujisawa, 252-0816, Japan

## Abstract

**Background:**

In the process of constructing a dynamic model of a metabolic pathway, a large number of parameters such as kinetic constants and initial metabolite concentrations are required. However, in many cases, experimental determination of these parameters is time-consuming. Therefore, for large-scale modelling, it is essential to develop a method that requires few experimental parameters. The hybrid dynamic/static (HDS) method is a combination of the conventional kinetic representation and metabolic flux analysis (MFA). Since no kinetic information is required in the static module, which consists of MFA, the HDS method may dramatically reduce the number of required parameters. However, no adequate method for developing a hybrid model from experimental data has been proposed.

**Results:**

In this study, we develop a method for constructing hybrid models based on metabolome data. The method discriminates enzymes into static modules and dynamic modules using metabolite concentration time series data. Enzyme reaction rate time series were estimated from the metabolite concentration time series data and used to distinguish enzymes optimally for the dynamic and static modules. The method was applied to build hybrid models of two microbial central-carbon metabolism systems using simulation results from their dynamic models.

**Conclusion:**

A protocol to build a hybrid model using metabolome data and a minimal number of kinetic parameters has been developed. The proposed method was successfully applied to the strictly regulated central-carbon metabolism system, demonstrating the practical use of the HDS method, which is designed for computer modelling of metabolic systems.

## Background

Since a biochemical network is essentially a nonlinear, nonequilibrium, non-steady-state system, dynamic simulation is especially effective for analyzing or predicting its behaviour in a detailed and realistic manner. However, a large amount of experimental information, including reaction mechanisms of enzymes, kinetic constants, and initial concentrations of enzymes and metabolites, is required to construct a dynamic model of a metabolic pathway. Although a number of high-throughput technologies for obtaining comprehensive biochemical data have been developed [1-6], most experimental methods for determining enzyme kinetics are of the low-throughput variety. Recently, several databases for enzyme kinetics have been published on the internet [7-9]. However, in many cases, the parameters in these databases are insufficient for building an accurate metabolic model. Moreover, although intracellular data can be collected from the published literature, experimental conditions and target strains are, in general, not uniform. Therefore, a huge amount of experimental work is currently needed to build an accurate dynamic model of a biochemical system. For this reason, a modelling method requiring less experimental effort needs to be developed.

Yugi *et al*. proposed a novel method for dynamic modelling of metabolism, the hybrid dynamic/static method (HDS method) [10]. The HDS method divides a dynamic system into a dynamic module and a static module. Enzyme reactions included in the dynamic module are represented by differential equations. Reaction rates of enzymes included in the static module are calculated by metabolic flux analysis (MFA) [11,12]. Since MFA needs no kinetic information, the amount of experimental work required is dramatically reduced. According to Okino and Mavrovouniotis's classification [13], the HDS method can be regarded as a "linear transformation into standard two-time-scale form," which is a time-scale analysis method. The superior points of the HDS method are its simple architecture and the admissibility of multiple metabolites. Only relationships among enzyme reactions are employed in the HDS method; thus a model builder does not have to consider the problem of multiple time-scale reactions of a given metabolite [14]. Since the Moore-Penrose pseudo-inverse [15,16] of the stoichiometric coefficient matrix for the unknown variables (*i.e. *reaction rates of enzymes in the static module) is applied in performing the MFA, the stoichiometric coefficient matrix for the unknown variables does not have to be square and regular.

Although the HDS method has the aforementioned advantages, no method has been proposed for splitting a dynamic system into a dynamic module and a static module before completion of the initial model construction. Advanced measurement technologies have been developed that now enable researchers to obtain the metabolome, that is, comprehensive metabolite concentration data [17-19]. It is reasonable to expect that the in-depth information of the metabolome contributes to the process for distinguishing dynamic and static enzymes in a metabolic system. In this study, we have developed a method of distinguishing dynamic and static enzymes based on metabolome data before construction of a complete model. The purpose of the proposed method is to provide the information (distinguishing dynamic from static enzymes) for initial HDS model construction required by the model builders without losing the advantage of the HDS method: reducing experimental efforts to obtain kinetic information of the modelled metabolic system. Identification of enzyme kinetic rate equations and the fitting of kinetic parameters using metabolite concentration data are outside the scope of this study. Moreover, biological meanings of the dynamic/static modules are not considered explicitly in the HDS method.

The proposed method consists of two parts. First, the enzyme reaction rate time series are estimated from metabolite concentration time series data. The dynamic and static enzymes are distinguished using the estimated enzyme reaction rate time series. The purpose of this study was to confirm that the proposed method can be used to construct accurate hybrid models, with accuracy comparable to that of a fully dynamic model. Therefore, we used pseudo-experimental data obtained from preliminarily constructed fully dynamic models. Two models of microorganisms, *Escherichia coli *[20] and *Saccharomyces cerevisiae *[21], were used for evaluation.

## Methods

### Hybrid dynamic/static method

The hybrid dynamic/static method (HDS method) is described in Yugi *et al. *[10]. Enzyme reaction rates in the static module are calculated by the following equation:

*v*_*static*_(*t*) = -*S*_*static*_^# ^· *S*_*module boundary *_· *v*_*module boundary*_(*t*)

where **v**_static _is the static module enzyme reaction rate vector, **v**_module boundary _is the module boundary enzyme reaction rate vector, **S**_static_^# ^is the Moore-Penrose pseudoinverse of the stoichiometric coefficient matrix for enzymes in the static module, and **S**_module boundary _is the stoichiometric coefficient matrix for module boundary enzymes. The HDS method aims to describe a system in which a quasi-steady state is attained in the static module at each instant, while the overall system (both the dynamic and the static modules) acts dynamically [10]. A transient value of the modelled system is calculated by an interaction between kinetic-based dynamic models and MFA-based static models.

### Estimation of internal enzyme reaction rates

**Figure 1:**
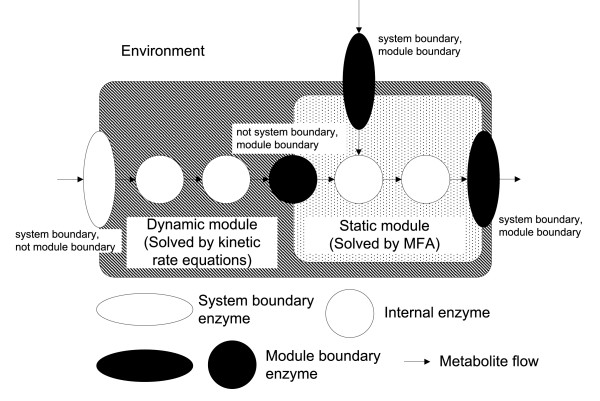
**Schematic diagram of hybrid model**. The hybrid model consists of a dynamic module (area shaded with diagonal lines) and a static module (dotted area). All module boundary enzymes should be included in the dynamic module. All system boundary enzymes are included in the dynamic module, but not all system boundary enzymes locate on the border between the static module and the dynamic module.

To calculate reaction rates of enzymes from metabolite concentrations, we define a "system boundary enzyme" as an enzyme located on the border of the metabolic system and extending outside the system. The system boundary enzyme is not the same as the "module boundary enzyme" defined by Yugi *et al. *[10]. A non system boundary enzyme is defined as an "internal enzyme." The relationship among the dynamic module, static module, module boundary enzyme, system boundary enzyme, and internal enzyme is shown in Figure 1. Since all system boundary enzymes should be included in the dynamic module, we assumed that the kinetics of system boundary enzymes have already been determined and that the reaction rates of system boundary enzymes can be calculated from metabolite concentrations.

The reaction rates of internal enzymes were calculated from the slopes of metabolite concentrations and the reaction rates of the system boundary enzymes. With the definitions of the system boundary enzyme and the internal enzyme, a mass balance equation of a metabolic system under a dynamic transition state can be expressed as follows [22]:

[S diag(−1)]⋅[vsystem boundary(t)vinternal(t)C′(t)]=O
 MathType@MTEF@5@5@+=feaafiart1ev1aaatCvAUfKttLearuWrP9MDH5MBPbIqV92AaeXatLxBI9gBaebbnrfifHhDYfgasaacH8akY=wiFfYdH8Gipec8Eeeu0xXdbba9frFj0=OqFfea0dXdd9vqai=hGuQ8kuc9pgc9s8qqaq=dirpe0xb9q8qiLsFr0=vr0=vr0dc8meaabaqaciaacaGaaeqabaqabeGadaaakeaadaWadaqaaiabdofatjabbccaGiabdsgaKjabdMgaPjabdggaHjabdEgaNjabcIcaOiabgkHiTiabigdaXiabcMcaPaGaay5waiaaw2faaiabgwSixpaadmaabaqbaeqabmqaaaqaaiabdAha2naaBaaaleaacqWGZbWCcqWG5bqEcqWGZbWCcqWG0baDcqWGLbqzcqWGTbqBcqqGGaaicqWGIbGycqWGVbWBcqWG1bqDcqWGUbGBcqWGKbazcqWGHbqycqWGYbGCcqWG5bqEaeqaaOGaeiikaGIaemiDaqNaeiykaKcabaGaemODay3aaSbaaSqaaGqaciab=LgaPjab=5gaUjab=rha0jabdwgaLjabdkhaYjabd6gaUjabdggaHjabdYgaSbqabaGccqGGOaakcqWG0baDcqGGPaqkaeaacuWGdbWqgaqbaiabcIcaOiabdsha0jabcMcaPaaaaiaawUfacaGLDbaacqGH9aqpieaacqGFpbWtaaa@6D1F@

where **S **is the stoichiometric coefficient matrix, diag(-1) is a diagonal matrix (column number = row number = metabolite number), **v**_system boundary_(*t*) is the system boundary enzyme reaction rate vector, **v**_internal_(*t*) is the internal enzyme reaction rate vector, and **C**'(*t*) is the metabolite concentration slope vector.

If **C**(*t*) and **v**_system boundary_(*t*) are known, the reaction rates of the internal enzymes can be estimated from Eq. (3), which is transformed from Eq. (2).

$$v_{internal}(t)=-S_{internal}^{\#}\cdot \left[S_{system\;boundary}\;diag(-1)\right]\cdot \left[\begin{array}{c} v_{system\;boundary}(t)\\ C^{\prime }(t) \end{array}\right]$$

where **S**_internal_^# ^is the Moore-Penrose pseudoinverse of the stoichiometric coefficient matrix for internal enzymes, and **S**_system boundary _is the stoichiometric coefficient matrix for system boundary enzymes [see Supplementary Text (see additional file 1) for an example of this procedure]. This procedure uses only the mass balance of the overall system and rate equations of the system boundary enzymes; thus, no information about regulation in the internal system is required beforehand. When Eq. (3) is applied to a determined system, the equation provides a true solution for **v**_internal_, and when Eq. (3) is applied to an over-determined system, the least-squares estimation of **v**_internal _is obtained [10]. In both cases, the solution is reasonable even if the modelled metabolic system has a complex network [10]. When Eq. (3) is applied to an under-determined system, the equation provides the least norm solution. However, such a least norm solution is not always a physiologically optimal estimation of **v**_internal_. This is a limitation of the current procedure.

### Evaluation of estimated internal enzyme reaction rates

The accuracy of the estimated internal enzyme reaction rates was evaluated by means of the reproduced metabolite concentration time series, which were calculated from the estimated enzyme reaction rates. Since it is difficult to compare the true and estimated reaction rates, we compared the metabolic concentrations. If an enzyme catalyzes a reversible reaction, the sign of the sum of the forward and reverse reaction rates may change. Near such a sign change, the calculated relative error between the true reaction rate and the estimated reaction rate may at times be a very large value (see Eq. (4) below). When the value of a data point is close to zero, a large error will be obtained. However, in general, most metabolite concentrations have a sufficiently large positive value for the problem caused by a value close to zero to be avoided.The metabolite concentration time series slope was calculated from the reaction stoichiometric matrix and each estimated enzyme reaction rate time series. The metabolite concentration time series was calculated by numerical integration of the metabolite concentration slope time series obtained. The mean relative error (MRE) [23] between the true values (data) and the calculated values in the metabolite concentration time series was calculated by the following equation:

MRE(%)=∑i=1nsamplingpoint∑j=1nmetabolite|Cdata,i,j−Cestimated,i,jCdata,i,j|nsamplingpoint⋅nmetabolite×100
 MathType@MTEF@5@5@+=feaafiart1ev1aaatCvAUfKttLearuWrP9MDH5MBPbIqV92AaeXatLxBI9gBaebbnrfifHhDYfgasaacH8akY=wiFfYdH8Gipec8Eeeu0xXdbba9frFj0=OqFfea0dXdd9vqai=hGuQ8kuc9pgc9s8qqaq=dirpe0xb9q8qiLsFr0=vr0=vr0dc8meaabaqaciaacaGaaeqabaqabeGadaaakeaacqWGnbqtcqWGsbGucqWGfbqrcqGGOaakcqGGLaqjcqGGPaqkcqGH9aqpdaWcaaqaamaaqahabaWaaabCaeaadaabdaqaamaalaaabaGaem4qam0aaSbaaSqaaiabdsgaKjabdggaHjabdsha0jabdggaHjabcYcaSiabdMgaPjabcYcaSiabdQgaQbqabaGccqGHsislcqWGdbWqdaWgaaWcbaGaemyzauMaem4CamNaemiDaqNaemyAaKMaemyBa0MaemyyaeMaemiDaqNaemyzauMaemizaqMaeiilaWIaemyAaKMaeiilaWIaemOAaOgabeaaaOqaaiabdoeadnaaBaaaleaacqWGKbazcqWGHbqycqWG0baDcqWGHbqycqGGSaalcqWGPbqAcqGGSaalcqWGQbGAaeqaaaaaaOGaay5bSlaawIa7aaWcbaGaemOAaOMaeyypa0JaeGymaedabaGaemOBa42aaSbaaWqaaiabd2gaTjabdwgaLjabdsha0jabdggaHjabdkgaIjabd+gaVjabdYgaSjabdMgaPjabdsha0jabdwgaLbqabaaaniabggHiLdaaleaacqWGPbqAcqGH9aqpcqaIXaqmaeaacqWGUbGBdaWgaaadbaGaem4CamNaemyyaeMaemyBa0MaemiCaaNaemiBaWMaemyAaKMaemOBa4Maem4zaCMaemiCaaNaem4Ba8gcbiGae8xAaKMae8NBa4Mae8hDaqhabeaaa0GaeyyeIuoaaOqaaiabd6gaUnaaBaaaleaacqWGZbWCcqWGHbqycqWGTbqBcqWGWbaCcqWGSbaBcqWGPbqAcqWGUbGBcqWGNbWzcqWGWbaCcqWGVbWBcqWFPbqAcqWFUbGBcqWF0baDaeqaaOGaeyyXICTaemOBa42aaSbaaSqaaiabd2gaTjabdwgaLjabdsha0jabdggaHjabdkgaIjabd+gaVjabdYgaSjabdMgaPjabdsha0jabdwgaLbqabaaaaOGaey41aqRaeGymaeJaeGimaaJaeGimaadaaa@B7F6@

where *C*_data,*i*,*j *_is the true concentration of the *j*-th metabolite at the *i*-th sampling point, *C*_estimated,*i*,*j *_is the estimated (reproduced) concentration of the *j*-th metabolite at the *i*-th sampling point, *n*_metabolite _is the number of metabolites, and *n*_sampling point _is the number of sampling points.

In this study, the MRE between the true metabolite concentration data and the reproduced metabolite concentrations is called the "basal error".

### Distinction of dynamic and static enzymes

**Figure 2:**
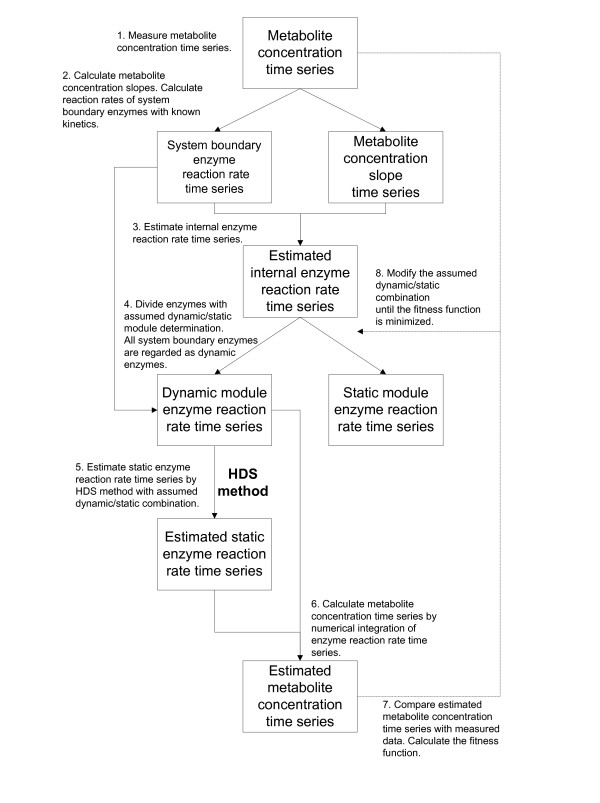
**Flowchart of distinguishing dynamic/static enzymes on the basis of metabolome data**. Simulation results from the dynamic models of *E. coli *and *S. cerevisiae *were used as pseudo experimental data to provide the metabolite concentrations required in the first step of the flowchart.

The genetic algorithm (GA) [24] is employed to search for an optimal dynamic/static enzyme combination in a metabolic system. In this work, an individual code set for the GA was defined to represent the dynamic/static enzymes in a metabolic system. For example, DDSSDD represents a metabolic system consisting of six enzymes: the 1st, 2nd, 5th, and 6th enzymes for the dynamic module and the 3rd and 4th enzymes for the static module. In the GA calculation, the enzyme reaction rate time series in the static module were calculated from enzyme reaction rate time series in the dynamic module, which were derived from metabolite concentration time series data, by the same HDS method. Consequently, each metabolite concentration time series data point was calculated by the same method as that described in "Evaluation of estimated internal enzyme reaction rates". The fitness function defined in Eq. (5) was calculated for each code set; thereafter, propagation, crossover, and mutation followed. This procedure was repeated until the optimal solution, which minimizes Eq. (5), was found. A flowchart of the process for distinguishing dynamic/static enzymes is shown in Figure 2.

f=∑i=1nsamplingpoint∑j=1nmetabolite(Cdata,i,j−Cestimated,i,jCdata,i,j)2nsamplingpoint⋅nmetabolite+w⋅(nenzyme−nstatic enzymenenzyme)2
 MathType@MTEF@5@5@+=feaafiart1ev1aaatCvAUfKttLearuWrP9MDH5MBPbIqV92AaeXatLxBI9gBaebbnrfifHhDYfgasaacH8akY=wiFfYdH8Gipec8Eeeu0xXdbba9frFj0=OqFfea0dXdd9vqai=hGuQ8kuc9pgc9s8qqaq=dirpe0xb9q8qiLsFr0=vr0=vr0dc8meaabaqaciaacaGaaeqabaqabeGadaaakeaacqWGMbGzcqGH9aqpdaWcaaqaamaaqahabaWaaabCaeaadaqadaqaamaalaaabaGaem4qam0aaSbaaSqaaiabdsgaKjabdggaHjabdsha0jabdggaHjabcYcaSiabdMgaPjabcYcaSiabdQgaQbqabaGccqGHsislcqWGdbWqdaWgaaWcbaGaemyzauMaem4CamNaemiDaqNaemyAaKMaemyBa0MaemyyaeMaemiDaqNaemyzauMaemizaqMaeiilaWIaemyAaKMaeiilaWIaemOAaOgabeaaaOqaaiabdoeadnaaBaaaleaacqWGKbazcqWGHbqycqWG0baDcqWGHbqycqGGSaalcqWGPbqAcqGGSaalcqWGQbGAaeqaaaaaaOGaayjkaiaawMcaamaaCaaaleqabaGaeGOmaidaaaqaaiabdQgaQjabg2da9iabigdaXaqaaiabd6gaUnaaBaaameaacqWGTbqBcqWGLbqzcqWG0baDcqWGHbqycqWGIbGycqWGVbWBcqWGSbaBcqWGPbqAcqWG0baDcqWGLbqzaeqaaaqdcqGHris5aaWcbaGaemyAaKMaeyypa0JaeGymaedabaGaemOBa42aaSbaaWqaaiabdohaZjabdggaHjabd2gaTjabdchaWjabdYgaSjabdMgaPjabd6gaUjabdEgaNjabdchaWjabd+gaVHqaciab=LgaPjab=5gaUjab=rha0bqabaaaniabggHiLdaakeaacqWGUbGBdaWgaaWcbaGaem4CamNaemyyaeMaemyBa0MaemiCaaNaemiBaWMaemyAaKMaemOBa4Maem4zaCMaemiCaaNaem4Ba8Mae8xAaKMae8NBa4Mae8hDaqhabeaakiabgwSixlabd6gaUnaaBaaaleaacqWGTbqBcqWGLbqzcqWG0baDcqWGHbqycqWGIbGycqWGVbWBcqWGSbaBcqWGPbqAcqWG0baDcqWGLbqzaeqaaaaakiabgUcaRiabdEha3jabgwSixpaabmaabaWaaSaaaeaacqWGUbGBdaWgaaWcbaGaemyzauMaemOBa4MaemOEaONaemyEaKNaemyBa0MaemyzaugabeaakiabgkHiTiabd6gaUnaaBaaaleaacqWGZbWCcqWG0baDcqWGHbqycqWG0baDcqWGPbqAcqWGJbWycqqGGaaicqWGLbqzcqWGUbGBcqWG6bGEcqWG5bqEcqWGTbqBcqWGLbqzaeqaaaGcbaGaemOBa42aaSbaaSqaaiabdwgaLjabd6gaUjabdQha6jabdMha5jabd2gaTjabdwgaLbqabaaaaaGccaGLOaGaayzkaaWaaWbaaSqabeaacqaIYaGmaaaaaa@DD54@

where *C*_data,*i*,*j *_is true concentration of the *j*-th metabolite at the *i*-th sampling point, *C*_estimated,*i*,*j *_is estimated concentration of the *j*-th metabolite at the *i*-th sampling point, *n*_metabolite _is number of metabolites, *n*_sampling point _is number of sampling points, *n*_enzyme _is number of internal enzymes, *n*_static enzyme _is number of enzymes included in static module, and *w *is weighting coefficient.

The first term in the fitness function represents the average error of the metabolite concentrations. For the fitness function, for the same reason as in the evaluation of estimated enzyme reaction rates, the metabolite concentrations rather than the enzyme reaction rates themselves were used. The second term in the fitness function evaluates the ratio of static enzymes included in the metabolic system; this term was added to adjust the number of enzymes in the static modules. The second term is multiplied by an adjusting parameter, a weighting coefficient, to control the balance between the model error and the static enzyme ratio.

9 different values of the weighting coefficient (*w *= 1.000, 0.750, 0.500, 0.250, 0.100, 0.075, 0.050, 0.025, and 0.010) were employed. The results of distinguishing dynamic and static enzymes were used to construct the hybrid models.

### Error calculation

MRE of the metabolite concentration time series in a result of the process for distinguishing dynamic/static enzymes or in a hybrid model was calculated by Eq. (4). Finally, in the process for distinguishing dynamic/static enzymes, the "basal error", which originated from the incompleteness of the estimation of the enzyme reaction rates and from the error of the numerical integration of the enzyme reaction rates, rather than from the HDS calculation, was subtracted from the MRE.

### Pseudo experiments

Two microbial central-carbon metabolism models were chosen for testing: the *E. coli *model constructed by Chassagnole *et al. *[20] and the *S. cerevisiae *model constructed by Hynne *et al. *[21]. For the *E. coli *model, starting from a steady state for which the extracellular glucose concentration was 5.56 × 10^-2 ^mM, a glucose pulse was added. The concentration of the injected glucose pulse was 1.67 mM. In Chassagnole's original model, time series of nucleotides (ATP/ADP/AMP, NAD(H), NADP(H)) were expressed by time-dependent functions [20]. However, in our study, the nucleotide concentrations were fixed as initial values. For the *S. cerevisiae *model, starting from a steady state for which the glucose concentration in the feed solution was 2.50 mM, the glucose concentration was shifted to 5.00 mM. The metabolite concentrations in both models at the steady state – that is, the initial concentrations for the dynamic simulations – are shown in Table S1 (see additional file 1). The running time after perturbation was set to 20 s for the *E. coli *model and 60 s for the *S. cerevisiae *model; these settings were chosen to allow time for the change from the original steady state to another steady state after the perturbation. The calculated metabolite concentration time series data sets were obtained at intervals of 1 s. These data sets were used as noise-free pseudo-metabolome data to calculate the slopes of the metabolite concentrations (**C**'(*t*)) and the reaction rates of the system boundary enzymes (**v**_system boundary_(*t*)) in a performance test of the method. The slopes of the metabolite concentrations were obtained by first-order differentiation of the interpolated metabolite concentration time series.

### Noise addition to the pseudo-experimental data

To evaluate the practical use of the proposed method, artificial noise was added to each pseudo-experimental metabolite concentration data point. The coefficient of variance (CV) was assumed to be 15%, and the standard deviation (SD) of each pseudo-experimental data point was calculated by multiplying the CV by the noise-free value. A normally distributed random number around the noise-free value was generated for each data point using the SD obtained. Five noise-added data points were generated for each noise-free data point as pseudo-replicated measurements. The average of the five noise-added data points was used in the following smoothing procedure.

### Smoothing of noisy pseudo-experimental data

Each noise-added metabolite concentration time series pseudo data set was smoothed by fitting it to a polynomial or a rational function of time using the least-squares method.

### Calculation tools

MATLAB Release 2006a (MathWorks) was used for all calculations. Ordinary differential equations were solved by the ODE15s algorithm [25]. For interpolation, differentiation and smoothing of the metabolite concentration time series data, Curve Fitting Toolbox 1.1.5 (MathWorks) was used. Cubic spline interpolation was employed. For optimization, the Genetic Algorithm and Direct Search Toolbox 2.0.1 (MathWorks) was employed. In each GA calculation, the number of code set was set to 100. The other parameters were set to default values. Each optimal solution was taken after the fitness function converged to a constant value.

## Results

### Estimation of enzyme reaction rates using noise-free data

In the HDS method, reaction rates of enzymes in a dynamic module are used to estimate reaction rates of enzymes in a static module. If the true reaction rates of all enzymes in a metabolic system are known, they can be used directly for discriminating dynamic and static enzymes. However, the true reaction rates of enzymes in a cell cannot be determined in most cases. Therefore, we tried to estimate the reaction rates of enzymes from metabolite concentrations, which can be experimentally measured by high-throughput metabolome technologies.

We calculated the estimated reaction rates by using the metabolite concentration time series obtained from the *E. coli *and *S. cerevisiae *models to evaluate our method of estimating reaction rates. In this section, the noise-free pseudo-experimental data were used to obtain a clear assessment of the estimation method itself. In the true reaction rate time series of Tkb in *E. coli*, TA in *E. coli*, and AK in *S. cerevisiae*, some sign-changing points were observed (Figure S1, see additional file 1). As predicted, around such points, huge relative errors between the true enzyme reaction rates and the estimated enzyme reaction rates were calculated (Figure S1). To avoid the undesired influence of such huge errors caused by using the reaction rates themselves, the reproduced metabolite concentrations were employed for the evaluation, as explained in the Methods. Therefore, the accuracy of the estimated reaction rates of the internal enzymes was assessed by the MRE between the original metabolite concentration time series and the reproduced metabolite concentration time series (Table 1). In the results for *E. coli*, the MRE was relatively large, mainly because of the large error in PGP. Errors in metabolites except for PGP were within approximately 10%; thus the estimation can be considered practically meaningful. For *S. cerevisiae*, errors of all metabolites were sufficiently small. On the whole, enzyme reaction rate time series data can be estimated from metabolite concentration time series data.

**Table 1:** Errors in reproduced metabolite concentrations obtained by using estimated enzyme reaction rates

*E. coli*		*S.cerevisiae*	
Metabolite	Error (%)	Metabolite	Error (%)

G6P	5.14 × 10^-1^	Glc	1.24 × 10^-1^
F6P	2.79	G6P	6.35 × 10^-2^
FDP	1.21	F6P	6.46 × 10^-2^
DHAP	2.16	FDP	2.38 × 10^-1^
GAP	1.95	DHAP	1.25 × 10^-1^
PGP	2.71 × 10^2^	GAP	1.40 × 10^-1^
3PG	1.01	PGP	3.36
2PG	5.88	PEP	6.88 × 10^-2^
PEP	8.27 × 10^-1^	Pyr	9.45 × 10^-2^
Pyr	2.45 × 10^-1^	ACA	3.91 × 10^-2^
6PG	2.06	EtOH	7.73 × 10^-3^
Ribu5P	1.04 × 10	Glyc	2.11 × 10^-2^
Xyl5P	8.08	ATP	5.67 × 10^-2^
Sed7P	9.06	ADP	3.36 × 10^-2^
Rib5P	2.91	AMP	1.28 × 10^-1^
E4P	7.40	NAD	3.74 × 10^-2^
G1P	2.82	NADH	1.18 × 10^-1^

MRE	1.94 × 10	MRE	2.77 × 10^-1^

### Distinction of dynamic and static enzymes using noise-free data

Using enzyme reaction rate time series data, we can apply the HDS method to calculate the reaction rates of static enzymes from the reaction rates of dynamic enzymes. These calculated static enzyme reaction rates can then be compared with the original reaction rate data. The errors between the estimated static enzyme reaction rates and the static enzyme reaction rate data can be used to find an optimal pattern for distinguishing dynamic from static enzymes. In this study, a fitness function (Eq. (5)) consisting of two terms was used for the optimization. In Eq. (5), the second term is multiplied by an adjusting parameter, a weighting coefficient (*w*). Even if the same data set is used, the result for distinguishing dynamic/static enzymes may vary for different *w*.

The *E. coli *and *S. cerevisiae *models and the estimated reaction rates obtained in the previous section (i.e., calculated from noise-free metabolite concentration data) were used to test this method for distinguishing enzymes, and the optimized patterns of dynamic and static enzymes shown in Table 2 were obtained as a result. As expected, the proportion of static enzymes decreased with decreasing *w*. The dynamic/static enzymes displayed on the metabolic map are shown in Supplementary Figure S2 (see additional file 1). The results obtained by using the noise-added metabolite concentration data are shown in the following section.

**Table 2:** Estimated patterns in distinguishing dynamic from static enzymes.

*E. coli*																		
w	1.000		0.750		0.500		0.250		0.100		0.075		0.050		0.025		0.010	

Noise	-	+	-	+	-	+	-	+	-	+	-	+	-	+	-	+	-	+

Fitness (-)	7.83 × 10^-1^	3.37	7.13 × 10^-1^	3.30	6.42 × 10^-1^	3.23	5.71 × 10^-1^	3.16	5.06 × 10^-1^	3.09	4.94 × 10^-1^	3.08	4.82 × 10^-1^	3.07	4.69 × 10^-1^	3.05	4.59 × 10^-1^	3.04

PGI	S	S	S	S	S	S	S	S	S	S	S	S	S	S	S	D	D	D
PFK	D	D	D	D	D	D	D	D	D	D	D	D	D	D	D	D	D	D
ALDO	D	D	D	D	D	D	D	D	D	D	D	D	D	D	D	D	D	D
TIS	S	S	S	S	S	S	S	S	S	S	S	S	S	S	S	S	S	S
GAPDH	D	D	D	D	D	D	D	D	D	D	D	D	D	D	D	D	D	D
PGK	D	D	D	D	D	D	D	D	D	D	D	D	D	D	D	D	D	D
PGluMu	D	D	D	D	D	D	D	D	D	D	D	D	D	D	D	D	D	D
ENO	D	D	D	D	D	D	D	D	D	D	D	D	D	D	D	D	D	D
PK	D	D	D	D	D	D	D	D	D	D	D	D	D	D	D	D	D	D
PGM	S	S	S	S	S	S	S	S	S	S	S	S	S	S	S	D	D	D
G6PDH	D	D	D	D	D	D	D	D	D	D	D	D	D	D	D	D	D	D
PGDH	D	D	D	D	D	D	D	D	D	D	D	D	D	D	D	D	D	D
Ru5P	S	S	S	S	S	S	S	S	S	S	S	S	S	S	S	S	S	D
R5PI	S	S	S	S	S	S	D	S	D	D	D	D	D	D	D	D	D	D
TKa	S	S	S	S	S	S	S	S	S	S	S	S	S	S	S	S	D	S
TKb	S	S	S	S	S	S	D	D	D	D	D	D	D	D	D	D	D	D
TA	S	S	S	S	S	S	S	S	D	D	D	D	D	D	D	S	D	S

*S. cerevisiae*																		

w	1000		0.750		0.500		0.250		0.100		0.075		0.050		0.025		0.010	

Noise	-	+	-	+	-	+	-	+	-	+	-	+	-	+	-	+	-	+

Fitness (-)	3.35 × 10^-1^	1.75 × 10^1^	2.64 × 10^-1^	1.75 × 10^1^	1.94 × 10^-1^	1.74 × 10^1^	1.10 × 10^-1^	1.73 × 10^1^	5.42 × 10^-2^	1.73 × 10^1^	4.17 × 10^-2^	1.73 × 10^1^	4.17 × 10^-2^	1.73 × 10^1^	1.68 × 10^-2^	1.72 × 10^1^	8.02 × 10^-3^	1.72 × 10^1^

PGI	D	D	D	D	D	D	D	D	D	D	D	D	D	D	D	D	D	D
PFK	D	D	D	D	D	D	D	D	D	D	D	D	D	D	D	D	D	D
ALDO	D	D	D	D	D	D	D	D	D	D	D	D	D	D	D	D	D	D
TIS	S	S	S	D	S	D	D	D	D	D	D	D	D	D	D	D	D	D
GAPDH	D	D	D	D	D	D	D	D	D	D	D	D	D	D	D	D	D	D
PGK	D	D	D	D	D	D	D	D	D	D	D	D	D	D	D	D	D	D
PGluMu	D	D	D	D	D	D	D	D	D	D	D	D	D	D	D	D	D	D
ENO	D	S	D	S	D	S	D	S	D	S	D	S	D	S	D	S	D	S
PK	D	D	D	D	D	D	D	D	D	D	D	D	D	D	D	D	D	D
PGM	S	S	S	S	S	S	S	S	S	S	S	S	S	S	S	S	D	S
G6PDH	D	D	D	D	D	D	D	D	D	D	D	D	D	D	D	D	D	D
PGDH	S	S	S	S	S	S	S	S	S	S	S	S	S	S	S	S	S	S
Ru5P	S	S	S	S	S	S	S	D	D	D	D	D	D	D	D	D	D	D
R5PI	S	S	S	S	S	S	S	S	S	S	S	S	S	S	S	S	D	D
TKa	S	S	S	S	S	S	S	S	S	S	S	S	S	S	S	S	S	S
TKb	S	S	S	S	S	S	S	S	D	S	D	S	D	S	D	S	D	S
TA	S	S	S	S	S	S	S	S	S	S	S	S	S	D	S	D	D	D

*w *is the weighting coefficient in the fitness function (Eq. (5)), and the symbols D and S denote enzymes in the dynamic and static modules, respectively. The system boundary enzymes were omitted from the table because all system boundary enzymes were represented as dynamic enzymes.

In the next step, the estimated optimal results for distinguishing dynamic/static enzymes in Table 2 were used to convert the full dynamic models for *E. coli *and *S. cerevisiae *to hybrid models. In a process for distinguishing dynamic/static enzymes – that is, numerical integration of a given enzyme reaction rate time-series curve – the calculated static enzyme reaction rates at one sampling point do not affect those calculated at the next sampling point. In contrast, in the HDS method – that is, the initial value problem of simultaneous differential equations – the calculated static enzyme reaction rates at one integration step affect the calculation in the next step. Accordingly, the error calculated in a process for distinguishing dynamic/static is not always equal to the error in the hybrid model. Thus, comparison of errors between these two types of calculations is required.

**Figure 3:**
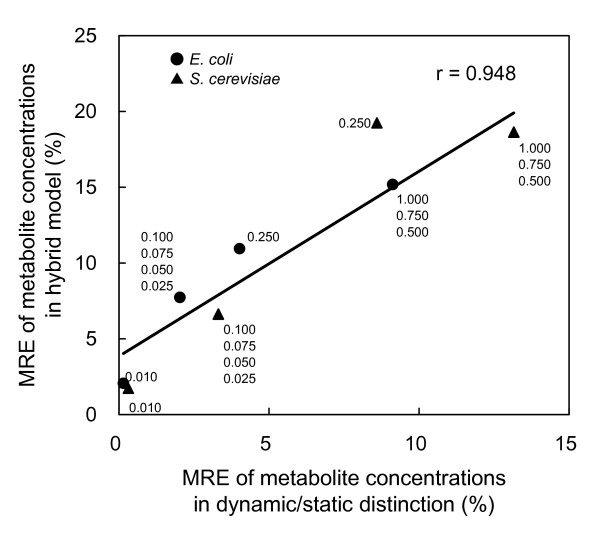
**Relationship of MRE of metabolite concentrations between processes for distinguishing dynamic/static enzymes and hybrid models**. The MRE s of the processes for distinguishing dynamic/static enzymes are the values after subtraction of the basal error (MRE shown in Table 1). Numbers next to the symbols represent weighting coefficients.

Figure 3 shows the relationship between the MRE of metabolite concentrations obtained by processes for distinguishing dynamic/static enzymes and the MRE of metabolite concentrations in the hybrid models for various weighting coefficients. The errors obtained by these two methods showed a high positive correlation (*r *= 0.948). This result indicates that the accuracy of the hybrid model constructed using the estimated distinguishing of dynamic/static enzymes exactly reflects the magnitude of the error estimated by processes for distinguishing dynamic/static enzymes. Therefore, the proposed method for distinguishing dynamic/static modules can be used to build a hybrid model.

**Figure 4:**
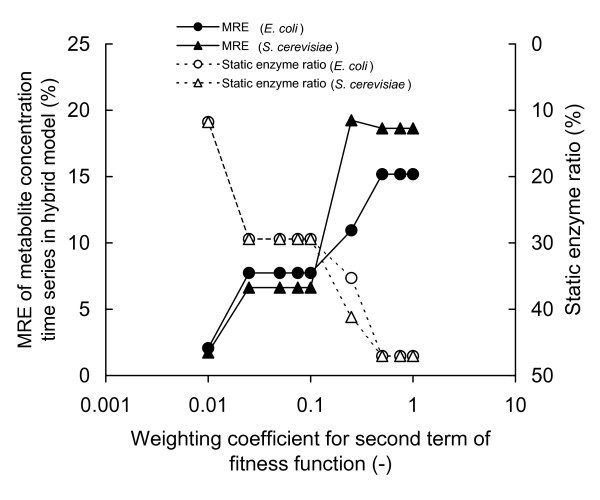
Relationships between *w *and MRE and *w *and the static enzyme ratio.

The error in the hybrid models was higher than that obtained by processes for distinguishing dynamic/static enzymes. In particular, in the distinguishing of dynamic/static enzymes of *S. cerevisiae *with *w *= 0.250, a considerable degree of error enlargement was shown in the hybrid model. This result can be considered to have been caused by error propagation at each integration step, as expected.The relationship between *w *and the MRE of the metabolite concentration time series and that between *w *and the static enzyme ratio was examined (Figure 4). The two metabolic systems tested showed very similar results, perhaps because both models deal with central-carbon metabolism. The dependency of the MRE and the static enzyme ratio on *w *showed a staircase pattern, rather than a pattern of simple linear increase (or decrease).

### Evaluation of the total process using noise-added data

In the previous sections, we used noise-free values to obtain a clear evaluation of the proposed method itself. However, real experimental data of metabolite concentrations are generally noisy. For practical use of the proposed method, the effect of noise on the process for distinguishing dynamic/static enzymes should be evaluated. Thus, we added noise to the noise-free data and then smoothed the noisy data for use in distinguishing the dynamic/static enzymes. In this study, simple smoothing by fitting to a polynomial or rational function of time was employed. The smoothing functions that were used and their parameters are shown in Supplementary Tables S2 and S3 (see additional file 1). Comparisons of noise-free values, noise-added values, and smoothed curves of metabolites are shown in Supplementary Figure S3 (see additional file 1). The results of distinguishing dynamic/static enzymes from the noisy metabolite concentration data are shown in Table 2. In most cases, when noise-added data were used, entirely or almost the same distinctions between dynamic/static enzymes were obtained as when noise-free data were used. However, in the results for *S. cerevisiae *obtained using smoothed noisy data, when *w *< 0.250, the number of static enzymes tended to be larger than in the results obtained using noise-free data. In the results for *E. coli*, the same tendency was observed when *w *= 0.010. Because the smoothing process of the metabolite concentration time series might result in loss of the high-frequency component of the time series data, the smoothed data might apparently change more slowly than is actually the case. Thus, when smoothed noisy data are used, the number of required dynamic enzymes in a HDS model tends to be smaller than the number needed when noise-free data are used. Because more precise metabolite concentrations need to be calculated when *w *is small, this tendency might be enhanced.

## Discussion

### Estimation of enzyme reaction rates

As shown in Table 1, the accuracy of the estimations of the enzyme reaction rates was confirmed by the reproduced metabolite concentrations, except for PGP in *E. coli*. Since the concentration of PGP was very low (average concentration, 3.60 × 10^-3 ^mM), even a slight error in the enzyme reaction rate had a large influence. In fact, the average errors between the true enzyme reaction rate time series and the estimated enzyme reaction rate time series for both GAPDH (PGP-producing enzyme) and PGK (PGP-consuming enzyme) in *E. coli *were adequately small, 2.44% and 1.46%, respectively. In the process for distinguishing dynamic/static enzymes, the average of the squared errors of all metabolite concentrations is used to calculate the fitness function (Eq. (5)); thus, an error in only one metabolite concentration has a limited effect. Actually, the results of distinguishing dynamic/static enzymes without the PGP time series (data not shown) were entirely the same as those shown in Table 2. However, if many metabolites with low concentrations are included in the modelled metabolic system, the processes for distinguishing dynamic/static enzymes may cause an erroneous conclusion to be drawn. This is a limitation of the current procedure. In comparison with the results for *E. coli*, errors for all metabolites for *S. cerevisiae *were adequately small, because the dynamics of the metabolic system in *S. cerevisiae *is relatively slow compared with the sampling frequency.

Another difficulty in applying the proposed method is that we assume that the concentrations of all metabolites are measurable. It is expected that high-throughput measurement techniques for detecting a huge number of metabolites, such as capillary electrophoresis combined with mass spectrometry (CE-MS) [17-19], can be used for such comprehensive measurements. The 1-s sampling interval employed in this study is feasible, because some rapid-sampling instruments capable of drawing multiple samples within 1 s from a bioreactor have already been developed [26-28].

### Distinction of dynamic and static enzymes

After a process for distinguishing dynamic/static enzymes is completed, the MRE in the corresponding hybrid model can be estimated using the linear relationship between the MRE in the process for distinguishing dynamic/static enzymes and the MRE in the hybrid model (Figure 3). This information helps to build a hybrid model that has the desired accuracy.

The staircase pattern of the relationships between the error and static enzyme ratio with decreasing *w*, observed in Figure 4, was probably caused by a property of metabolic systems. In a testing system, the number of enzymes that can potentially be allocated to the static module may be restricted. If *w *is greatly changed, the few potentially static enzymes would eventually start to be converted to static enzymes.

### Weighting coefficient in the fitness function

The weighting coefficient in the fitness function (Eq. (5)) is a tuning parameter. Since a suitable value for the weighting coefficient (*w*) is not given *a priori*, we need to consider how to define the value.

As shown in Figure 4, with a *w *of 1.000, about half of the enzymes were discriminated to the static module. Thus, a large amount of experimental work can be saved because no kinetic information is required by the static module. The MRE at *w *= 1.000 was 15.2% for the *E. coli *hybrid model and 18.6% for the *S. cerevisiae *hybrid model (Figure 4). These errors are acceptable considering the accuracy of the experimentally measured metabolite concentrations. Thus, *w *= 1.000 may simply be chosen at the initial trial stage of model construction. When a more precise model is required, a smaller *w *can be used. Even if *w *is set to between 0.025 and 0.100, the proportion of static enzymes remains at about 30% for both the metabolic systems tested. Our recommendation for *w *for general modelling is 0.050. At around this *w *value, the sensitivity of the error to a change of *w *is low; thus, strict specification of *w *is not required. Moreover, even if the actual error in the constructed hybrid model becomes considerably higher than the expected value *– *as in the case of *S. cerevisiae *at *w *= 0.250 *–*the actual error remains low.

### Noise in metabolome data

As shown in Table 2, almost the same results in distinguishing dynamic/static enzymes were obtained between the procedures using noise-free data and those using noise-added data. This result could be predicted because most metabolite time series were successfully reproduced from the noisy data by the smoothing treatment, as shown in Figure S3. This result indicates that the proposed method for distinguishing dynamic/static enzymes can be applied to noisy measurements if a suitable noise reduction method is employed. To remove noise and obtain the slopes of metabolite concentration time series, a smoothing technique based on an artificial neural network, proposed by Voit *et al. *[29-31], is efficient. Many other noise cancellation techniques have been proposed for biochemical time series data [32-35]. For example, Rizzi *et al. *[36] obtained time-course functions of metabolites from noisy metabolite concentration measurements and used those functions to tune the parameters in their dynamic model.

### Toward construction of accurate hybrid models

In the HDS method, accurate kinetics should be known not only for system boundary enzymes but also for all enzymes assigned to the dynamic modules. For this reason, high-throughput techniques for determining accurate and detailed enzyme kinetics are needed for the efficient development of models of metabolic systems. A promising power-law approach, generalized mass action (GMA) [37,38], may be used to solve this problem. This method has a large representational space that enables enzyme kinetics to be sufficiently expressed in spite of its simple fixed form. Although modelling that uses this kind of power-law approach from time series data is often difficult owing to their nonlinear properties, Polisetty *et al. *[39] have proposed a method employing branch-and-bound principles to find optimized parameters in GMA models. Using this method, the global optimal parameter set can be efficiently searched.

To ensure the validity of the predicting performance of an HDS model, careful perturbation experiments should be carried out to obtain the metabolome time series data to be used for distinguishing dynamic/static enzymes. The metabolite concentration variations used should be those considered to be of the maximum possible magnitude under the modelled conditions. To reproduce a rapidly changing metabolite concentration time series by an HDS model, a larger number of dynamic enzymes is required. Thus, if the number of dynamic enzymes included in the model is defined by using data showing the maximum possible variation in magnitude, that is, the model is constructed with the maximum possible number of dynamic enzymes, then the model can calculate all probable states of the system. For instance, consider building a metabolic model of cultured cells in a reactor, where the model has no mechanism for calculating gene expression levels or the consequent changes in protein concentrations (most proposed metabolic models are of this type). A substrate-pulse injection experiment giving the maximal substrate concentration that does not cause changes in gene expression levels in the cells (i.e., enzyme concentrations in the cells are kept constant) is useful for distinguishing dynamic/static enzymes. To determine the maximal permitted substrate concentration, many preliminary experiments may be required, and this seems to decrease the value of the HDS method, which aims to reduce experimental efforts. However, fundamentally speaking, such evaluation of the limits of a model's parameters is absolutely necessary for maintaining the accuracy of calculations in any kind of modelling, not only in HDS modelling. Therefore, this requirement for experiments to determine the maximal possible variation is not a specific disadvantage of the HDS method.

## Conclusion

The proposed method of using metabolite concentration time series,*i.e*., experimentally measurable variables, enables us to discriminate dynamic/static enzymes to construct a hybrid model. In this method, the enzyme reaction rate time series are estimated from metabolite concentration time series data. Since this estimation relies on only the mass balance in the system, no kinetic information about internal enzymes is required. Therefore, the aim of employing the HDS method – to reduce the experimental effort required to obtain enzyme kinetics information – can be achieved. Two microbial central-carbon metabolism models were used to evaluate our method. Central-carbon metabolism has many feedback loops and is rigidly controlled to maintain homeostasis of a living cell. Since our method was successfully applied for such a strictly regulated system, we believe it will have wide-ranging applicability to many types of metabolic systems. Furthermore, the analysis using noisy metabolite concentration data demonstrated that, for the most part, the proposed method tolerates noise well.

## Abbreviations

Metabolites

2PG 2-phosphoglycerate

3PG 3-phosphoglycerate

6PG 6-phosphogluconate

ACA acetaldehyde, intracellular

ACA_x _acetaldehyde, extracellular

CN_o _cyanide, mixed flow

CN_x _cyanide, extracellular

DHAP dihydroxyacetone phosphate

E4P erythrose 4-phosphate

EtOH ethanol, intracellular

EtOH_x _ethanol, extracellular

F6P fructose 6-phosphate

FDP fructose 1,6-bisphosphate

G1P glucose 1-phosphate

G6P glucose 6-phosphate

GAP glyceraldehyde 3-phosphate

Glc_o _glucose, mixed flow

Glc_x _glucose, extracellular

Glyc glycerol, intracellular

Glyc_x _glycerol, extracellular

PEP phosphoenolpyruvate

PGP 1,3-bisphosphoglycerate

Pyr pyruvate

Rib5P ribose 5-phosphate

Ribu5P ribulose 5-phosphate

Sed7P sedoheptulose 7-phosphate

Xyl5P xylulose 5-phosphate

Enzymes/reactions

ADH acetaldehyde dehydrogenase

AK adenylate kinase

ALDO aldolase

consum ATP consumption

difACA diffusion of acetaldehyde

difEtOH diffusion of EtOH

difGlyc diffusion of glycerol

ENO enolase

G6PDH glucose-6-phosphate dehydrogenase

GAPDH glyceraldehyde-3-phosphate dehydrogenase

GlcTrans glucose transporter

HK hexokinase

lpGlyc lumped glycerol formation reaction

lpPEP lumped PEP formation reaction

PDC pyruvate decarboxylase

PFK phosphofructokinase

PGDH 6-phosphogluconate dehydrogenase

PGI glucose-6-phosphate isomerase

PGK phosphoglycerate kinase

PGluMu phosphoglycerate mutase

PGM phosphoglucomutase

PK pyruvate kinase

R5PI ribose-phosphate isomerase

Ru5P ribulose-phosphate epimerase

TA transaldolase

TIS triosephosphate isomerase

TKa transketolase, reaction a

TKb transketolase, reaction b

## Competing interests

The author(s) declare that they have no competing interests.

## Authors' contributions

NI contributed to the development of the proposed method and wrote this manuscript. YN provided the basic ideas and directed the project, and MT was the project leader. All authors read and approved the final manuscript.

## Supplementary Material

Additional file 1 Supplementary information for "Distinguishing enzymes using metabolome data for the hybrid dynamic/static method". An example of the estimation of internal enzyme reaction rates (supplementary text), supplementary tables for conditions of simulation and smoothing (Table S1, Table S2, and Table S3) and supplementary figures of results (Figure S1, Figure S2 and Figure S3). Click here for file
