# Supplementary material for: Distinguishing enzymes using metabolome data for the hybrid dynamic/static method
Source: Theor Biol Med Model. 2007 May 20;4:19. doi: 10.1186/1742-4682-4-19 (PMC1892778; doi:10.1186/1742-4682-4-19)
Supplement: Additional file 1 — Supplementary information for "Distinguishing enzymes using metabolome data for the hybrid dynamic/static method". An example of the estimation of internal enzyme reaction rates (supplementary text), supplementary tables for conditions of simulation and smoothing (Table S1, Table S2, and Table S3) and supplementary figures of results (Figure S1, Figure S2 and Figure S3). [file 1742-4682-4-19-S1.pdf]

Supplementary Text 1 An example of the estimation of internal enzyme reaction rates.

An example of a small system is shown to explain how to calculate internal enzyme reaction rates from metabolite concentrations and reaction rates of system boundary enzymes. Consider a metabolic system consisting of three metabolites and three enzymes:

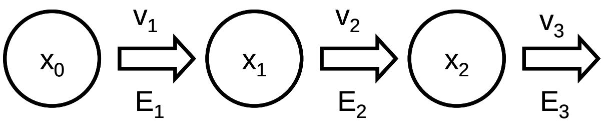

where x_0, x_1, and x_2 are metabolites; E_1, E_2, and E_3 are enzymes; and v_1, v_2, and v_3 are enzyme reaction rates. The mass balance of this system can be expressed by the following simultaneous ordinary differential equations:

$$\frac{dx_0}{dt} = -v_1$$
$$\frac{dx_1}{dt} = v_1 - v_2$$
$$\frac{dx_2}{dt} = v_2 - v_3$$

Accordingly, the overall system can be described by the following equation:

$$\begin{bmatrix} -1 & 0 & 0 & -1 & 0 & 0 \\ 1 & -1 & 0 & 0 & -1 & 0 \\ 0 & 1 & -1 & 0 & 0 & -1 \end{bmatrix} \begin{bmatrix} v_1 \\ v_2 \\ v_3 \\ \frac{dx_0}{dt} \\ \frac{dx_1}{dt} \\ \frac{dx_2}{dt} \end{bmatrix} = \mathbf{O}$$

In this system, E3 is a system boundary enzyme. When the kinetics of E3 is known and all metabolite concentrations are measurable, this equation can be separated to an unknown-variables (v_1 and v_2) part and a known-variables part:

$$\begin{bmatrix} -1 & 0 \\ 1 & -1 \\ 0 & 1 \end{bmatrix} \cdot \begin{bmatrix} v_1 \\ v_2 \end{bmatrix} + \begin{bmatrix} 0 & -1 & 0 & 0 \\ 0 & 0 & -1 & 0 \\ -1 & 0 & 0 & -1 \end{bmatrix} \cdot \begin{bmatrix} v_3 \\ \frac{dx_0}{dt} \\ \frac{dx_1}{dt} \\ \frac{dx_2}{dt} \end{bmatrix} = \mathbf{O}$$

Thus, the equation to estimate v_1 and v_2 can be written as follows:

$$\begin{bmatrix} v_1 \\ v_2 \end{bmatrix} = -\begin{bmatrix} -1 & 0 \\ 1 & -1 \\ 0 & 1 \end{bmatrix}^{\#} \cdot \begin{bmatrix} 0 & -1 & 0 & 0 \\ 0 & 0 & -1 & 0 \\ -1 & 0 & 0 & -1 \end{bmatrix} \cdot \begin{bmatrix} v_3 \\ \frac{dx_0}{dt} \\ \frac{dx_1}{dt} \\ \frac{dx_2}{dt} \end{bmatrix}$$

# denotes the Moore-Penrose pseudo-inverse. By substituting values of v_3, dx_0/dt, dx_1/dt, and dx_2/dt at each sampling point into the above equation, time series of v_1 and v_2 can be obtained.

Table S1 Initial metabolite concentrations.

The metabolite concentrations marked with asterisks were constant at the initial values.

A. *E. coli*

Metabolite	Concentration (mM)
Glc_x	5.56×10^{-2}
G6P	3.48
F6P	6.00×10^{-1}
FDP	2.72×10^{-1}
DHAP	1.67×10^{-1}
GAP	2.18×10^{-1}
PGP	8.00×10^{-3}
3PG	2.13
2PG	3.99×10^{-1}
PEP	2.67
Pyr	2.67
6PG	8.08×10^{-1}
Ribu5P	1.11×10^{-1}
Xyl5P	1.38×10^{-1}
Sed7P	2.76×10^{-1}
Rib5P	3.98×10^{-1}
E4P	9.80×10^{-2}
G1P	6.53×10^{-1}
AMP*	9.55×10^{-1}
ADP*	5.95×10^{-1}
ATP*	4.27
NADP*	1.95×10^{-1}
NADPH*	6.20×10^{-2}
NAD*	1.47
NADH*	1.00×10^{-1}

B. *S. cerevisiae*

Metabolite	Concentration (mM)
Glc_o^*	2.50
Glc_x	3.33×10^{-2}
Glc	3.71×10^{-3}
G6P	5.71×10^{-1}
F6P	7.20×10^{-2}
FDP	5.09×10^{-2}
DHAP	2.85×10^{-1}
GAP	1.24×10^{-2}
PGP	3.13×10^{-5}
PEP	6.27×10^{-3}
Pyr	6.54×10^{-2}
ACA	1.27×10^{-1}
ACA_x	1.10×10^{-1}
EtOH	3.75
$EtOH_x$	3.21
Glyc	3.64×10^{-1}
$Glyc_x$	1.46×10^{-1}
CN_x	5.56
CN_o^*	5.60
AMP	6.04×10^{-1}
ADP	1.76
ATP	1.57
NAD	7.79×10^{-1}
NADH	2.01×10^{-1}

Table S2 Smoothing functions applied to noisy metabolite concentration time series.

Name	Formula
poly1	$C(t) = p_1 \cdot t + p_2$
poly2	$C(t) = p_1 \cdot t^2 + p_2 \cdot t + p_3$
poly3	$C(t) = p_1 \cdot t^3 + p_2 \cdot t^2 + p_3 \cdot t + p_4$
poly4	$C(t) = p_1 \cdot t^4 + p_2 \cdot t^3 + p_3 \cdot t^2 + p_4 \cdot t + p_5$
poly5	$C(t) = p_1 \cdot t^5 + p_2 \cdot t^4 + p_3 \cdot t^3 + p_4 \cdot t^2 + p_5 \cdot t + p_6$
rat11	$C(t) = \dfrac{p_1 \cdot t + p_2}{t + p_3}$
rat22	$C(t) = \dfrac{p_1 \cdot t^2 + p_2 \cdot t + p_3}{t^2 + p_4 \cdot t + p_5}$
rat31	$C(t) = \dfrac{p_1 \cdot t^3 + p_2 \cdot t^2 + p_3 \cdot t + p_4}{t + p_5}$

$C(t)$, metabolite concentration time series (mM); t, time (s) ; p_n, n-th parameter of the time function.

Table S3 Parameters of smoothing functions applied to noisy metabolite concentration time series.

(A)

Metabolite	Glc_x	G6P	F6P	FDP	DHAP	GAP	PGP	3PG	2PG
Formula	poly1	rat22	rat22	rat22	rat22	rat22	rat31	rat22	rat31
p_1	-2.0118×10^{-3}	3.5917	6.1787×10^{-1}	1.2581	3.8058×10^{-1}	4.4714×10^{-1}	-2.8616×10^{-5}	1.0841	-2.3092×10^{-3}
p_2	1.6109	-1.4389×10	-9.1722×10^{-2}	-4.8399×10^{-1}	-6.3025×10^{-1}	-3.1038×10^{-1}	1.0654×10^{-3}	-8.3869	8.7585×10^{-2}
p_3		2.3019×10	3.0122×10^{-1}	4.1135	1.7377	9.3203×10^{-1}	-9.1496×10^{-3}	3.4353×10	-8.6563×10^{-1}
p_4		-4.0590	-3.9900×10^{-1}	-4.1372	-3.2714	-1.4055	6.3836×10^{-2}	-3.2100	4.8161
p_5		6.0083	5.3084×10^{-1}	1.3659×10	9.1551	4.1901	7.0708	1.7223×10	1.0895×10

(B)

Metabolite	PEP	Pyr	6PG	Ribu5P	Xyl5P	Sed7P	Rib5P	E4P	G1P
Formula	rat22	rat11	poly2	rat22	rat22	rat11	rat11	rat11	poly2
p_1	1.3117	4.2778	-1.0415×10^{-3}	1.3188×10^{-1}	1.7556×10^{-1}	1.9147×10^{-1}	5.1540×10^{-1}	1.6910×10^{-1}	-1.8735×10^{-4}
p_2	-5.6198	1.4626	2.1053×10^{-2}	-5.2550×10^{-1}	-1.0527×10^{-1}	1.3057	2.3579×10^{-1}	8.3945×10^{-2}	-5.9819×10^{-4}
p_3	6.3349×10	5.7016×10^{-1}	7.8927×10^{-1}	6.2064×10^{-1}	1.2366×10^{-1}	4.9895	6.4828×10^{-1}	1.0058	6.5185×10^{-1}
p_4	4.3019			-4.2332	-8.4976×10^{-1}				
p_5	2.2247×10			5.1292	8.9735×10^{-1}				

Table S3 (Continued.)

(C)

Metabolite	Glcx	Glc	G6P	F6P	FDP	DHAP	GAP	PGP	PEP
Formula	rat11	poly5	poly2	poly2	poly5	poly4	poly4	poly3	poly4
p_1	7.9886×10^{-2}	4.7503×10^{-10}	-2.9131×10^{-4}	-3.2491×10^{-5}	-1.0649×10^{-8}	-6.2214×10^{-7}	-3.5786×10^{-8}	5.2856×10^{-10}	-9.2207×10^{-9}
p_2	1.5901×10^{-2}	-8.5157×10^{-8}	2.6422×10^{-2}	3.0782×10^{-3}	1.1537×10^{-6}	9.2409×10^{-5}	5.1155×10^{-6}	-7.3373×10^{-8}	1.3130×10^{-6}
p_3	4.1942×10^{-1}	5.5718×10^{-6}	5.8214×10^{-1}	7.4694×10^{-2}	-1.9499×10^{-5}	-4.6791×10^{-3}	-2.4786×10^{-4}	3.2859×10^{-6}	-6.3740×10^{-5}
p_4		-1.5717×10^{-4}			-1.3878×10^{-3}	8.8994×10^{-2}	4.4396×10^{-3}	2.5254×10^{-5}	1.1917×10^{-3}
p_5		1.6347×10^{-3}			4.4834×10^{-2}	1.7593×10^{-1}	7.3898×10^{-3}		4.9841×10^{-3}
p_6									

(D)

Metabolite	Pyr	ACA	ACA_x	EtOH	$EtOH_x$	Glyc	$Glyc_x$	CN_x	ATP
Formula	poly4	poly3	poly3	poly4	poly2	poly4	poly4	poly2	rat22
p_1	-7.2655×10^{-8}	1.9992×10^{-6}	1.6963×10^{-6}	4.4071×10^{-7}	-2.7646×10^{-4}	7.0661×10^{-8}	5.3770×10^{-8}	-1.0099×10^{-4}	1.7557
p_2	1.1154×10^{-5}	-2.4707×10^{-4}	-2.0826×10^{-4}	-5.4620×10^{-5}	4.6656×10^{-2}	-9.4961×10^{-6}	-7.8714×10^{-6}	3.5016×10^{-3}	-5.3278×10
p_3	-5.9903×10^{-4}	9.7003×10^{-3}	8.4974×10^{-3}	1.6849×10^{-3}	3.0583	2.8632×10^{-4}	3.7106×10^{-4}	5.6207	5.0301×10^{2}
p_4	1.2867×10^{-2}	1.2358×10^{-1}	9.4787×10^{-2}	3.4301×10^{-2}		7.1687×10^{-3}	-3.6804×10^{-3}		-3.2783×10
p_5	5.6075×10^{-2}			3.6968		3.5349×10^{-1}	1.5741×10^{-1}		3.3690×10^{2}
p_6									

Table S3 (Continued.)

(E)

Metabolite	ADP	AMP	NAD	NADH
Formula	poly5	poly5	rat22	poly5
p_1	1.3700×10^{-8}	2.7583×10^{-8}	8.1936×10^{-1}	3.2000×10^{-9}
p_2	-2.8300×10^{-6}	-4.9102×10^{-6}	-1.9233×10	-7.6100×10^{-7}
p_3	2.0570×10^{-4}	3.1757×10^{-4}	2.1257×10^{2}	6.6000×10^{-5}
p_4	-6.1334×10^{-3}	-8.7275×10^{-3}	-2.2396×10	-2.5037×10^{-3}
p_5	5.7833×10^{-2}	8.1022×10^{-2}	2.8200×10^{2}	3.6732×10^{-2}
p_6				

(A) and (B), *E. coli*; (C), (D), and (E), *S. cerevisiae*. Formulas are shown in Table S2.

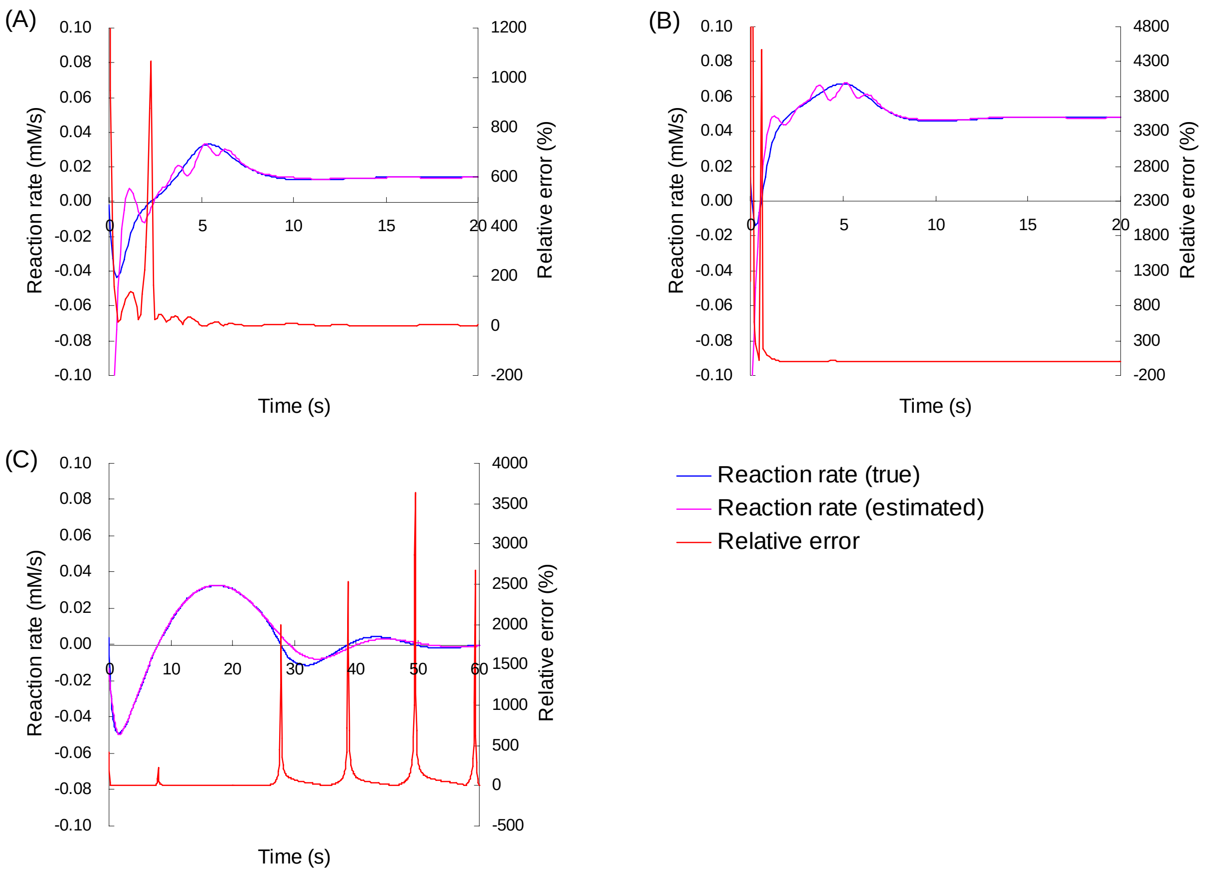

Figure S1 Relative error between true and estimated values of the enzyme reaction rate.
(A) *E. coli*, TKb; (B) *E. coli*, TA; (C) *S. cerevisiae*, AK.

(A)

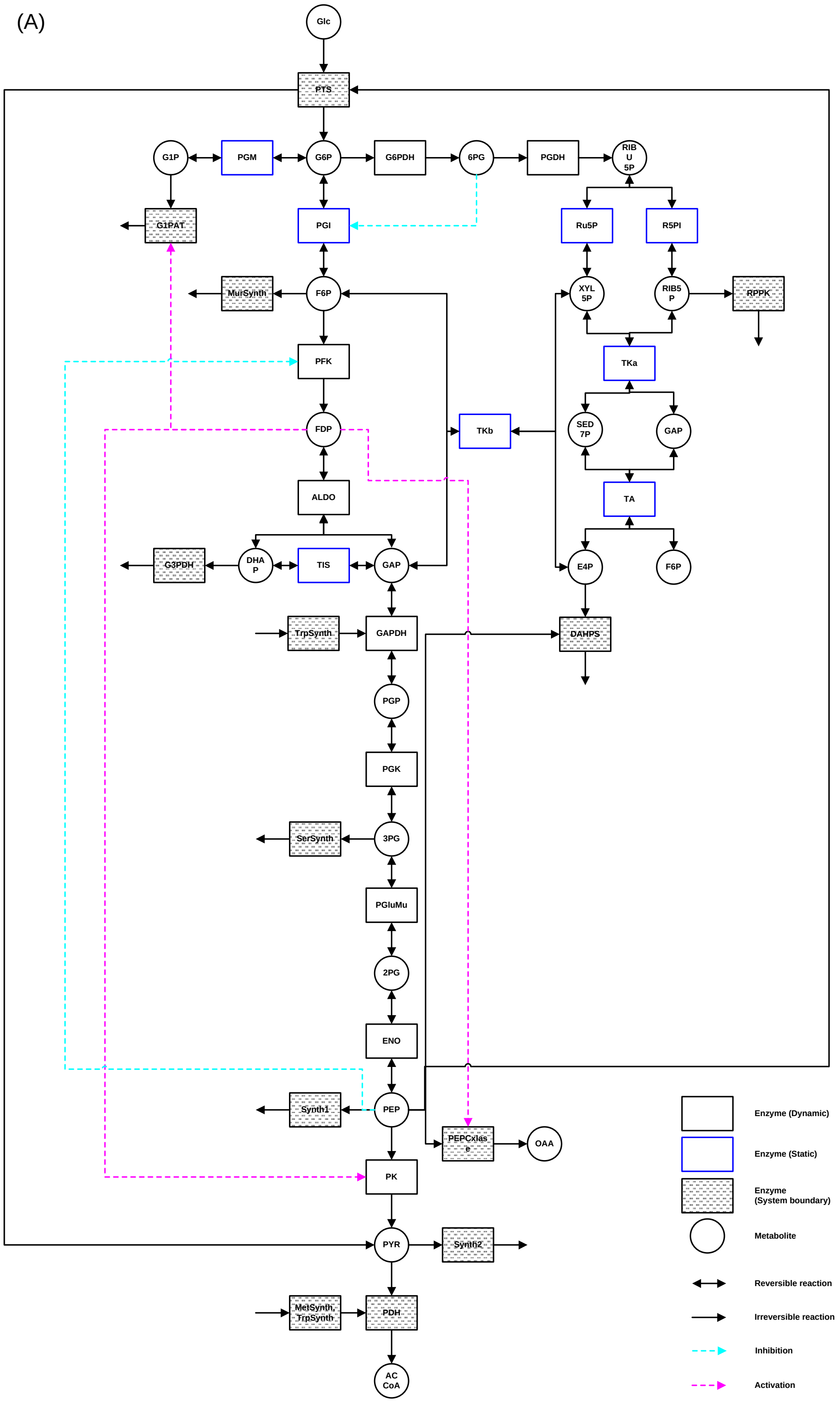
Glc
PTS
G1P
PGM
G6P
G6PDH
6PG
PGDH
RIB U 5P
G1PAT
PGI
Ru5P
R5PI
MurSynth
F6P
XYL 5P
RIB5 P
RPPK
PFK
TKa
FDP
TKb
SED 7P
GAP
ALDO
TA
G3PDH
DHA P
TIS
GAP
E4P
F6P
TrpSynth
GAPDH
DAHPS
PGP
PGK
SerSynth
3PG
PGluMu
2PG
ENO
Synth1
PEP
PEPCxlase
OAA
PK
PYR
Synth2
MetSynth, TrpSynth
PDH
AC CoA
Enzyme (Dynamic)
Enzyme (Static)
Enzyme (System boundary)
Metabolite
Reversible reaction
Irreversible reaction
Inhibition
Activation

Figure S2

(B)

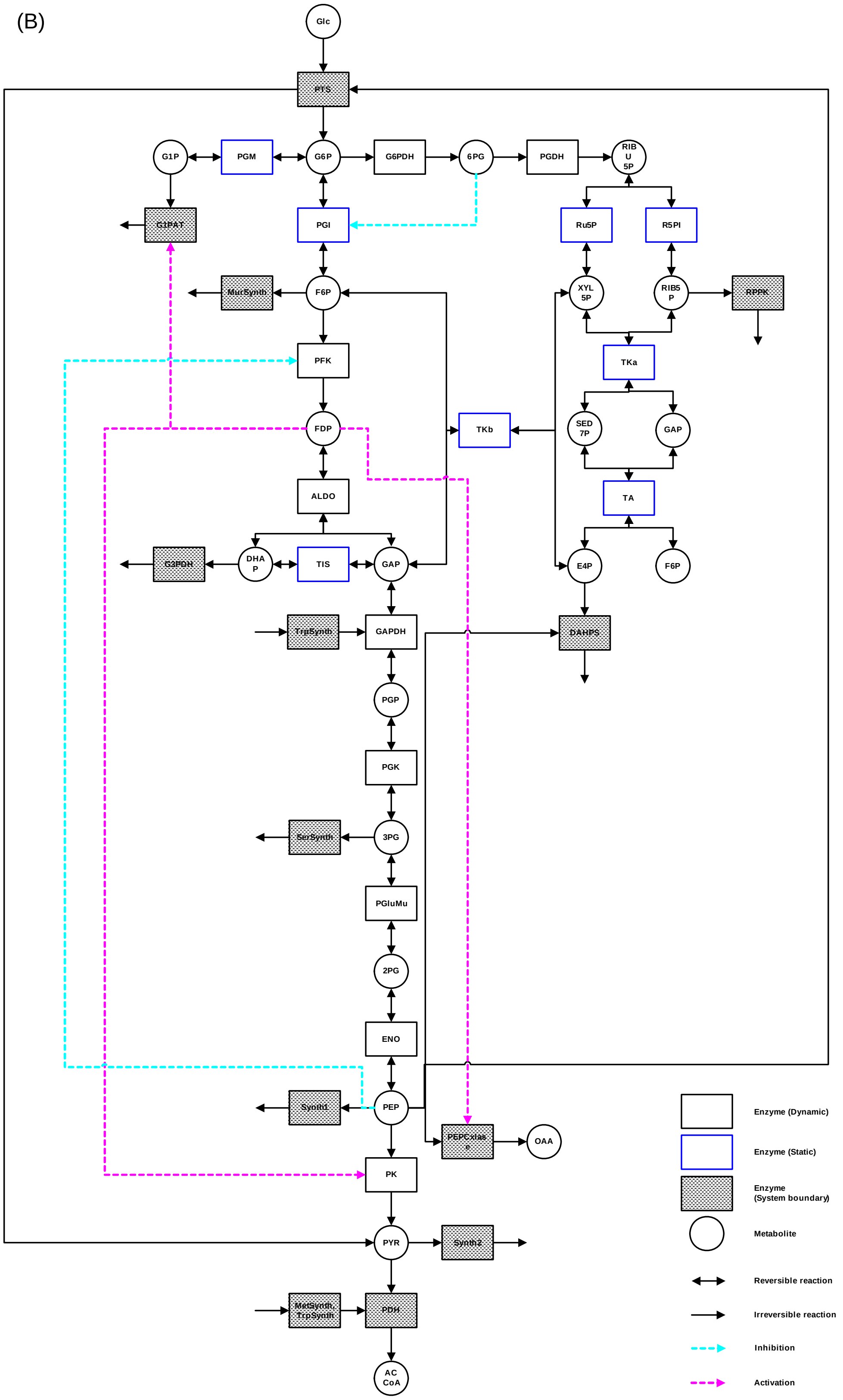

Figure S2 (Continued.)

(C)

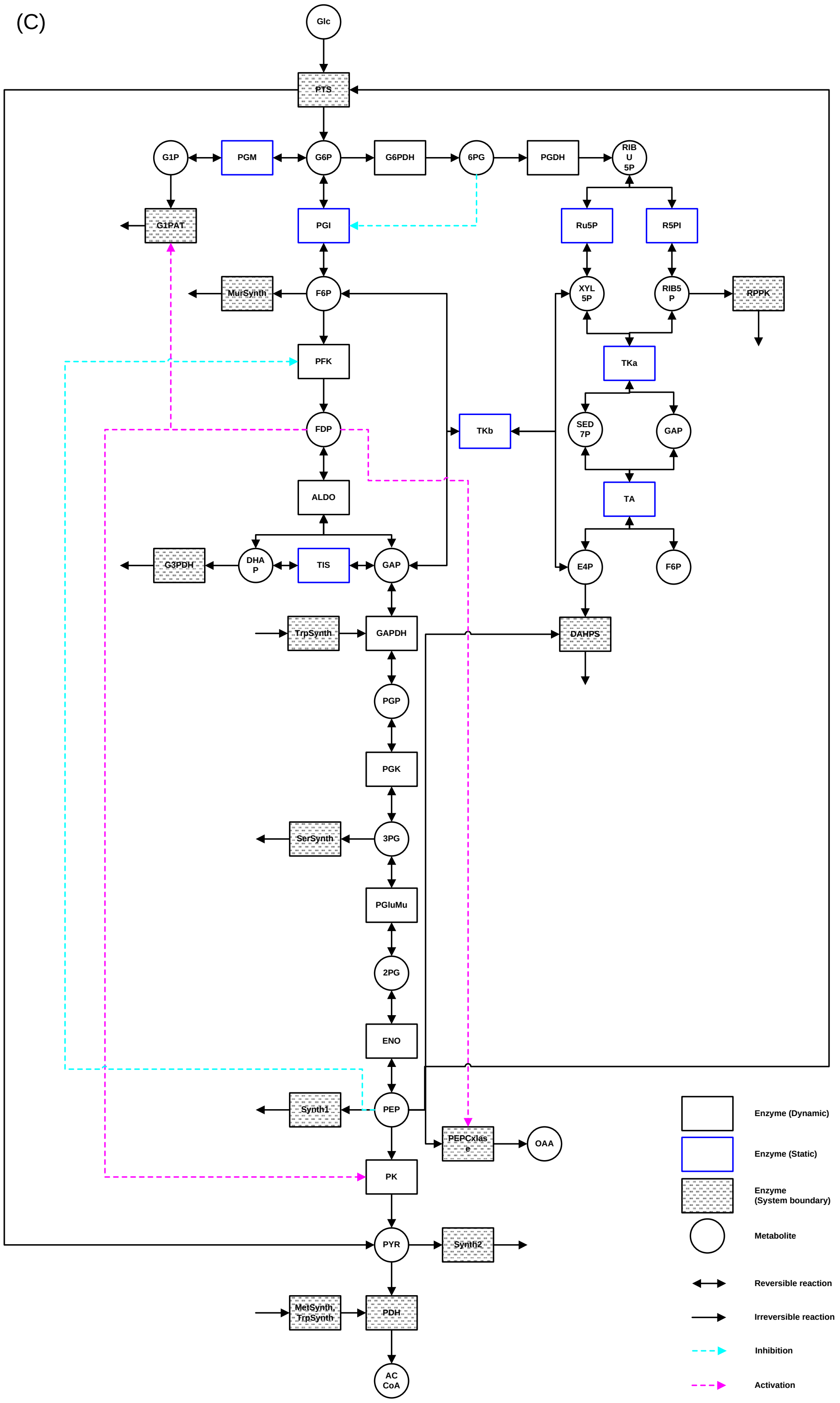

Figure S2 (Continued.)

(D)

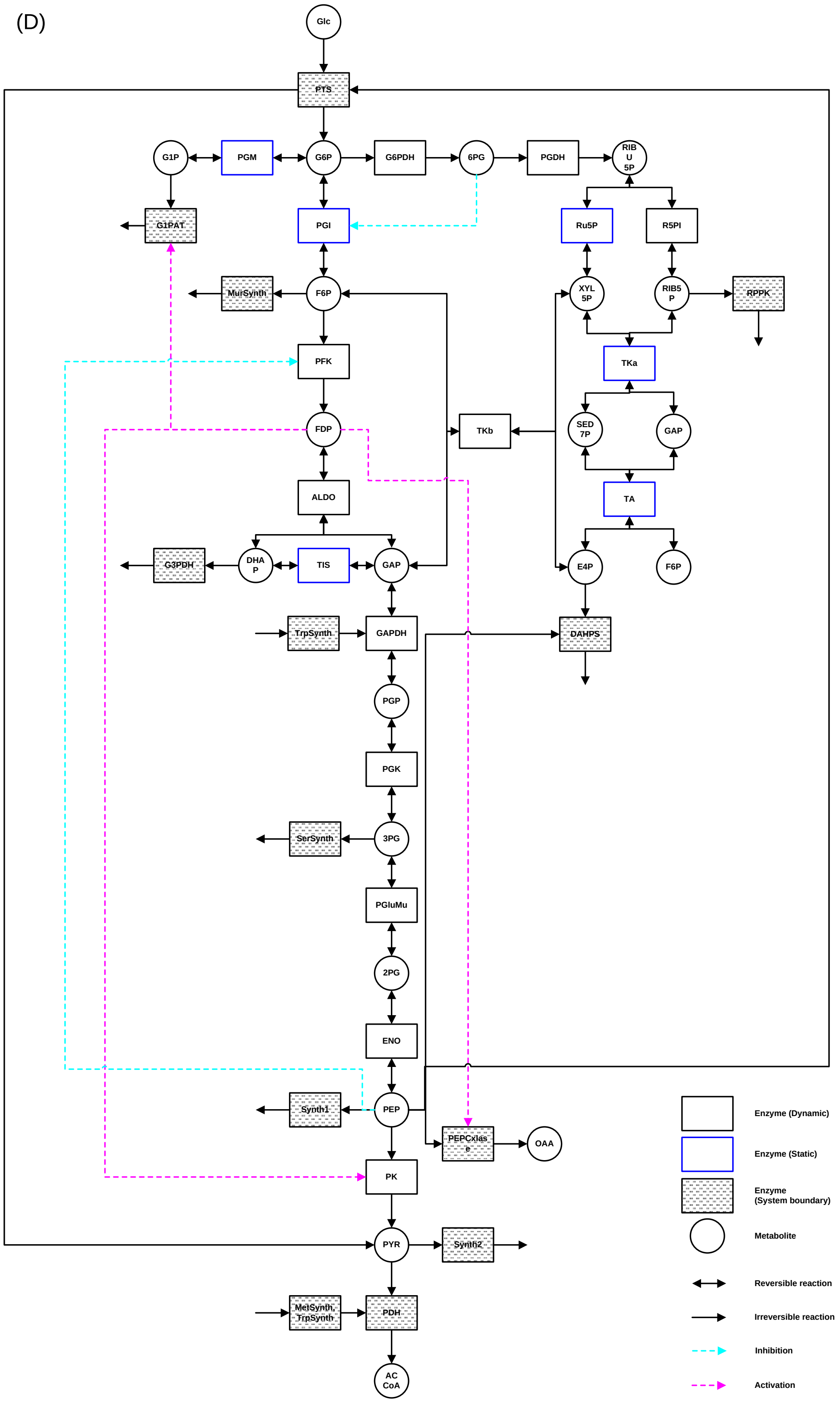

Figure S2 (Continued.)

(E)

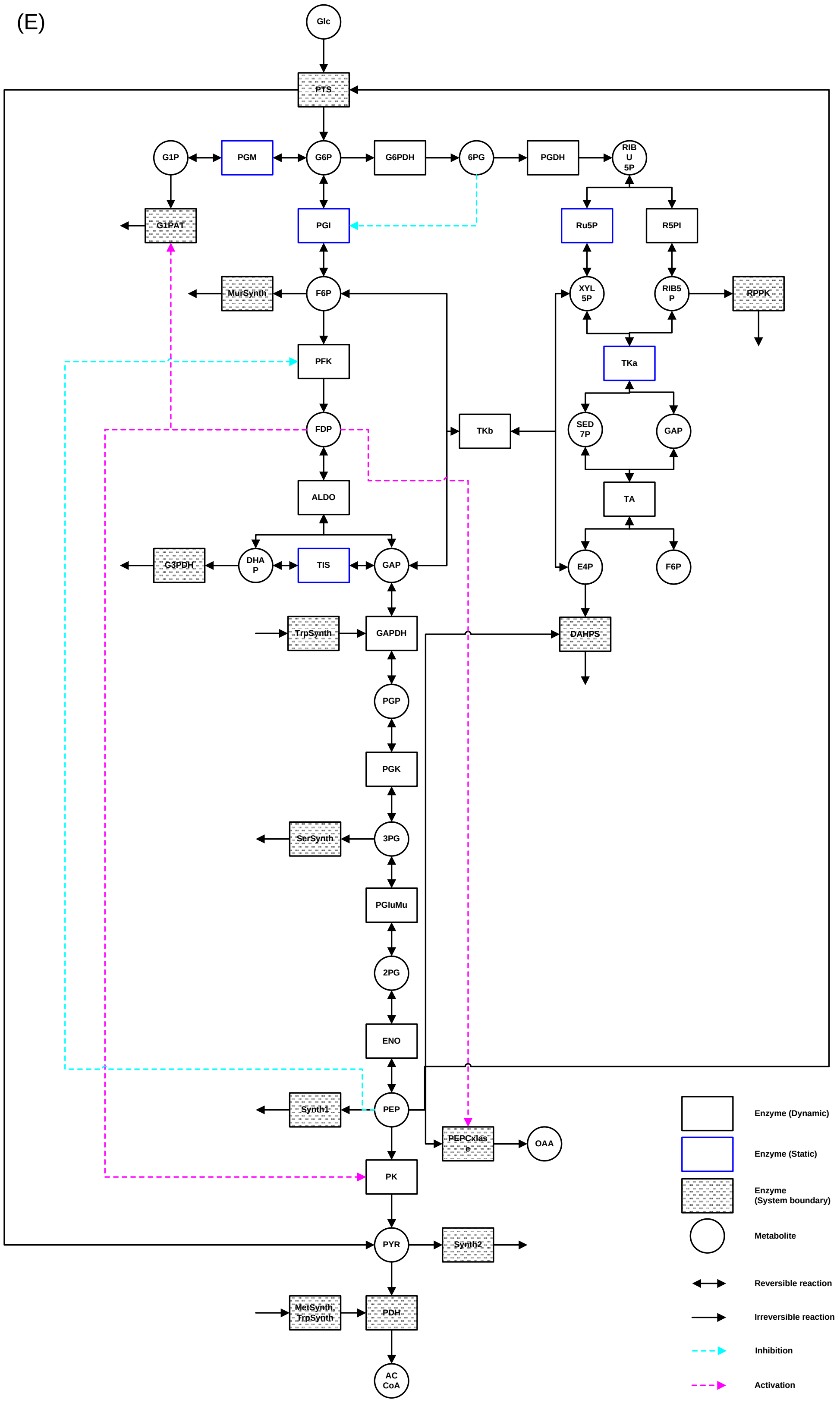

Figure S2 (Continued.)

(F)

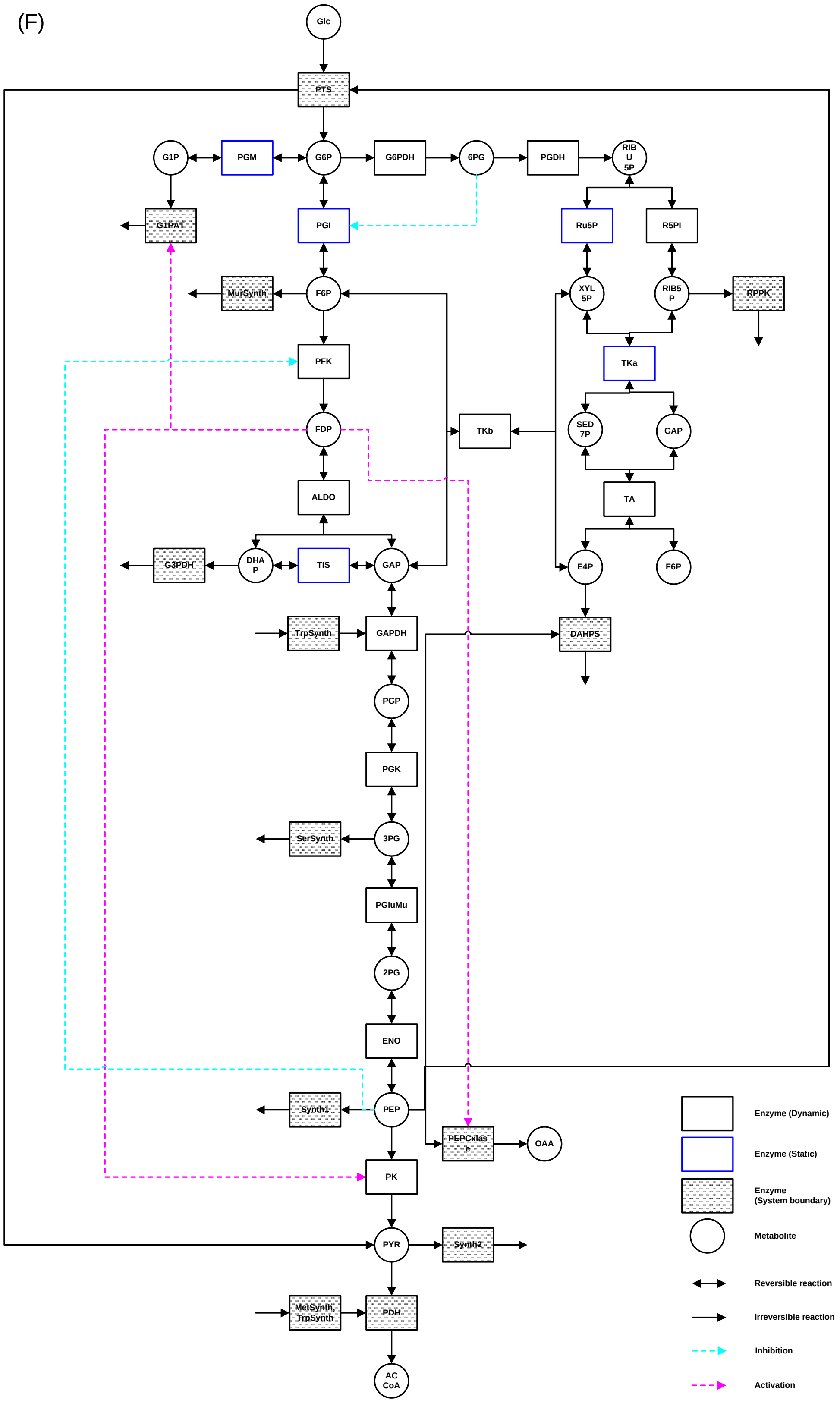

Figure S2 (Continued.)

(G)

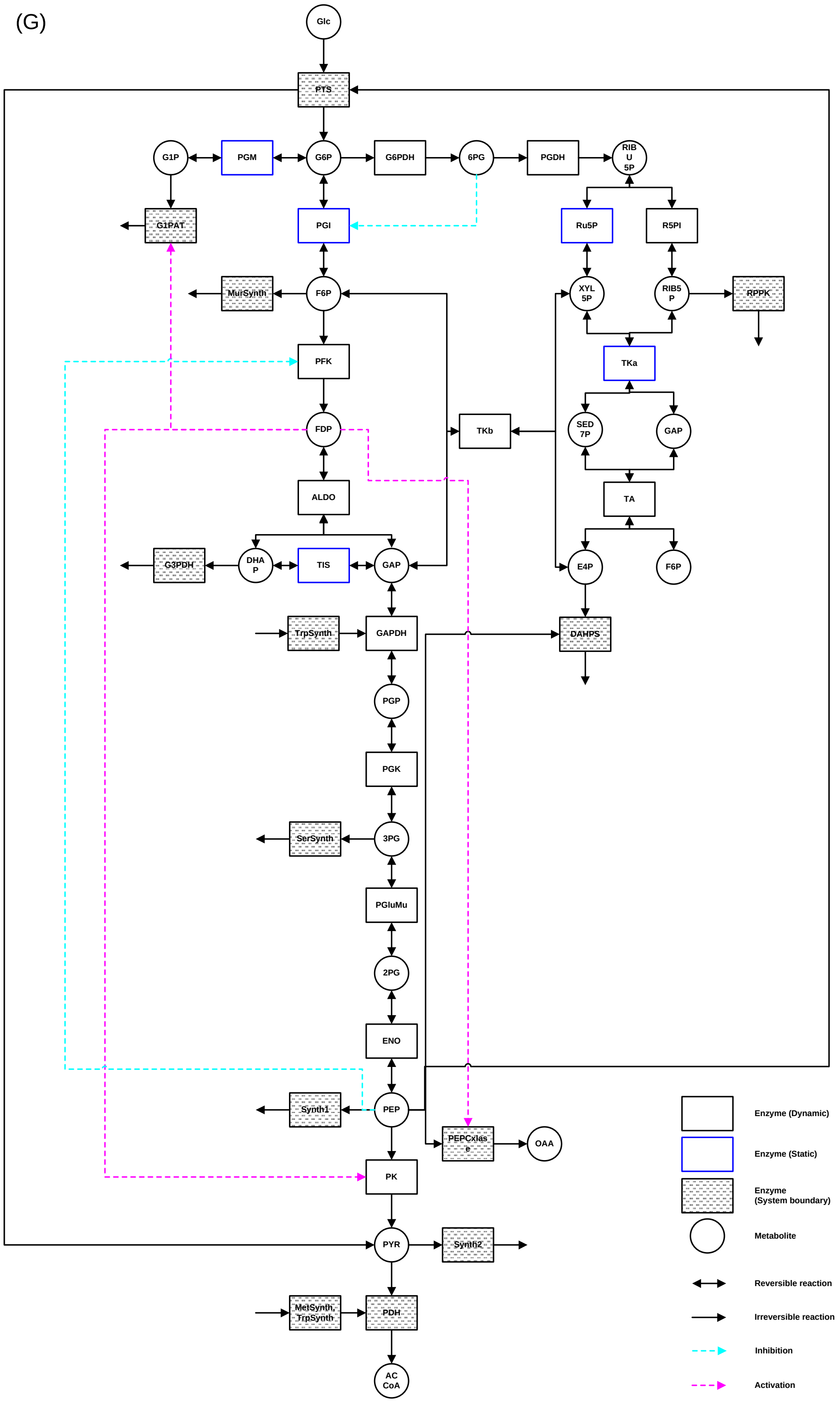

Figure S2 (Continued.)

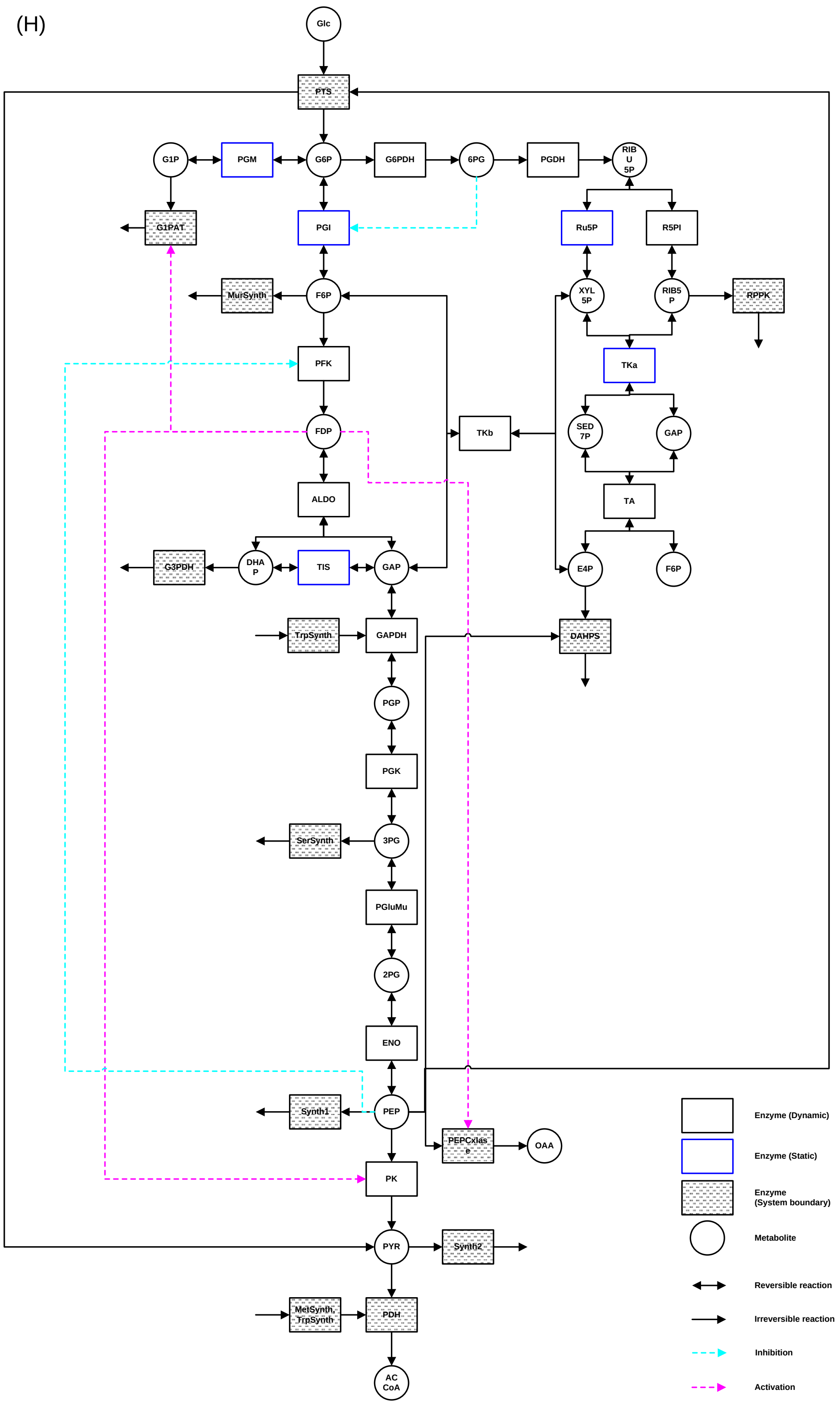

Figure S2 (Continued.)

(I)

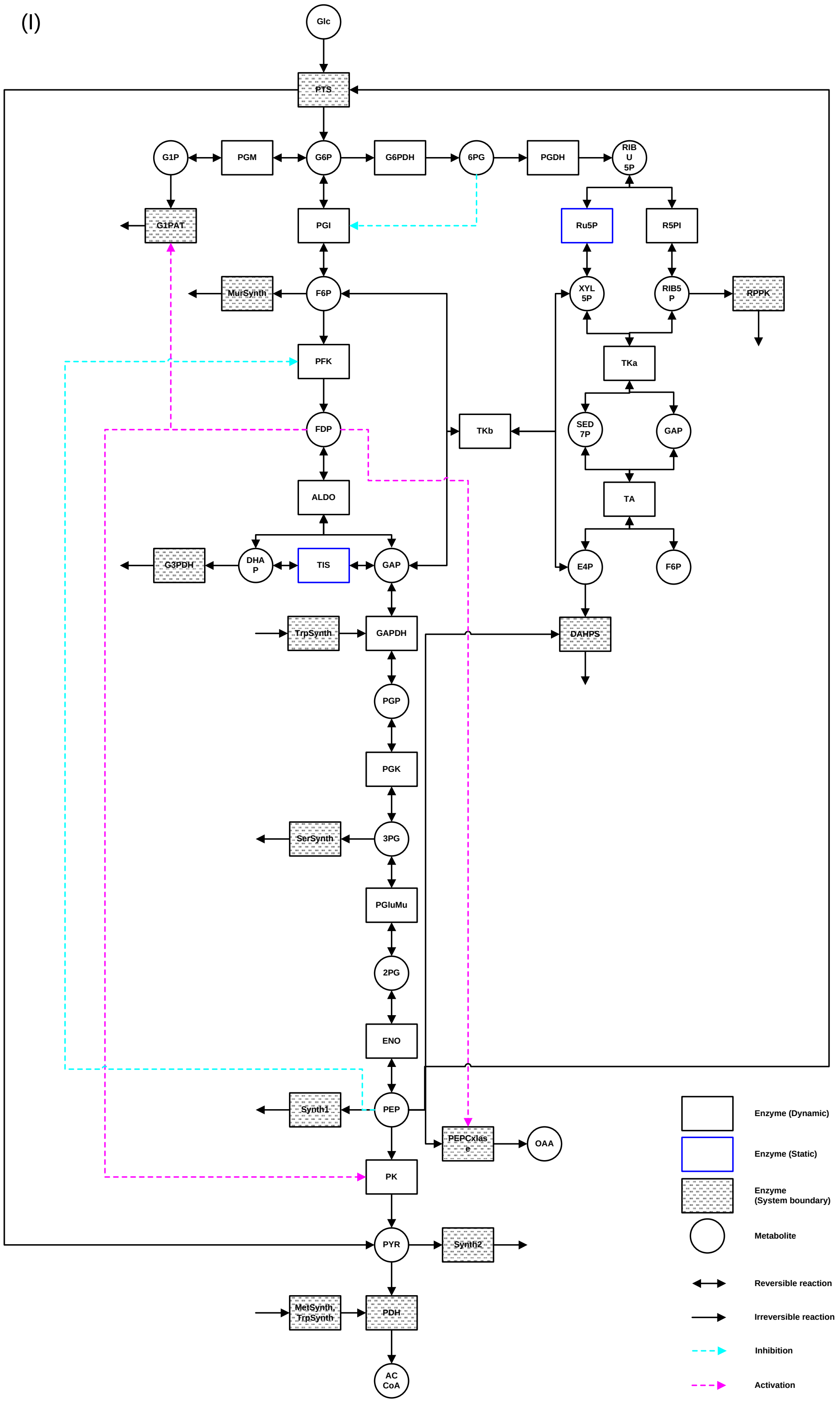

Figure S2 (Continued.)

(J)

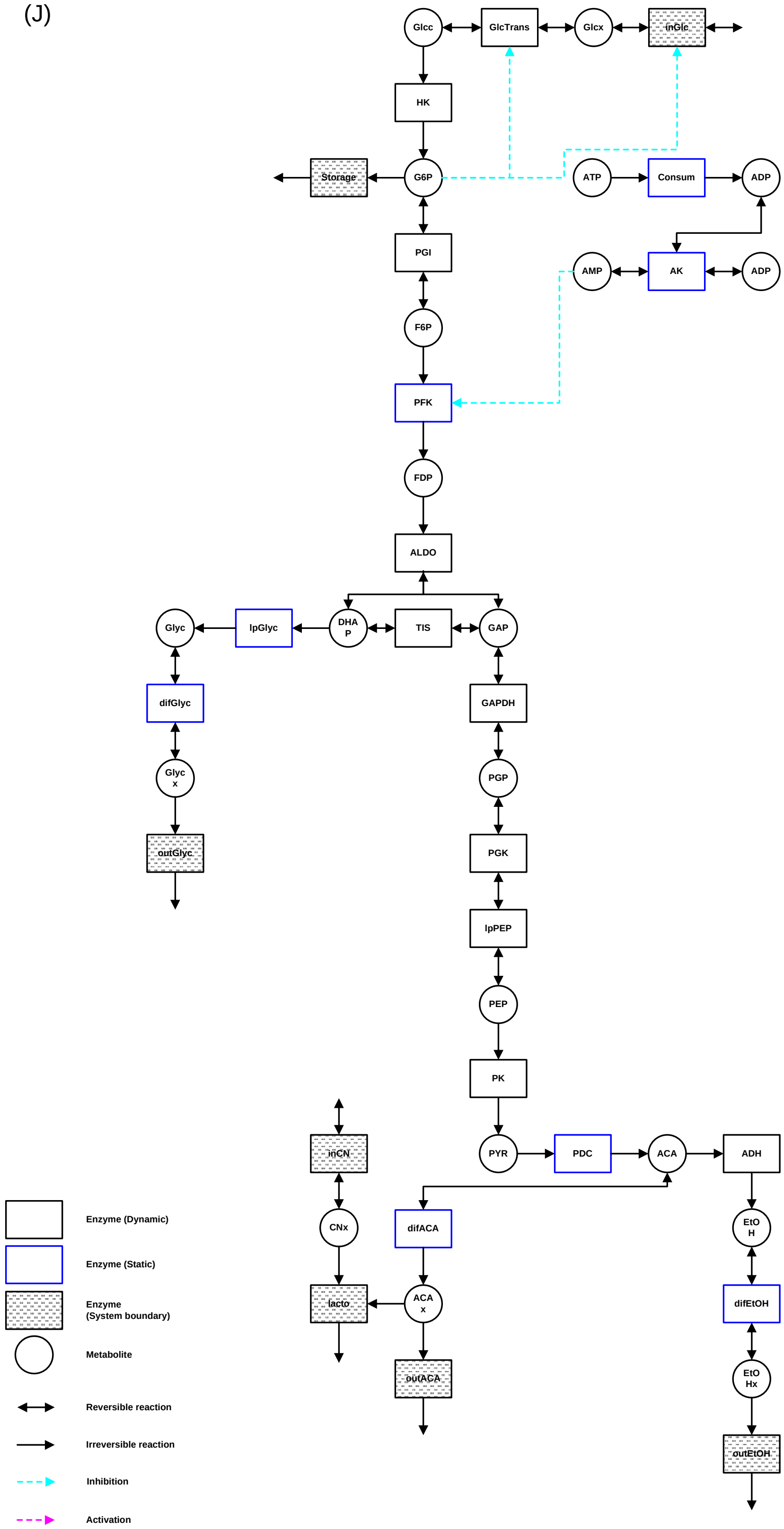

Figure S2 (Continued.)

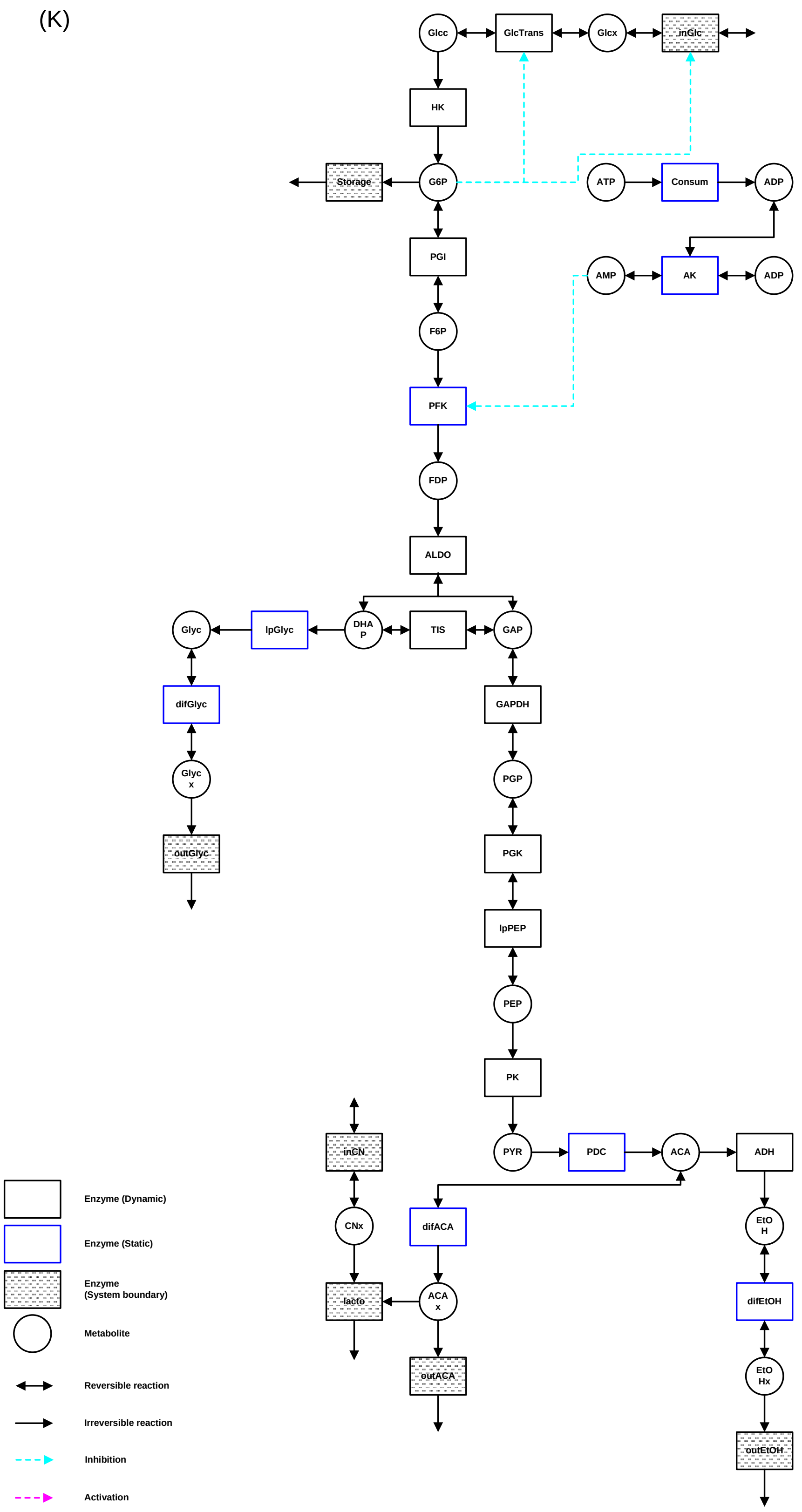

Figure S2 (Continued.)

(L)

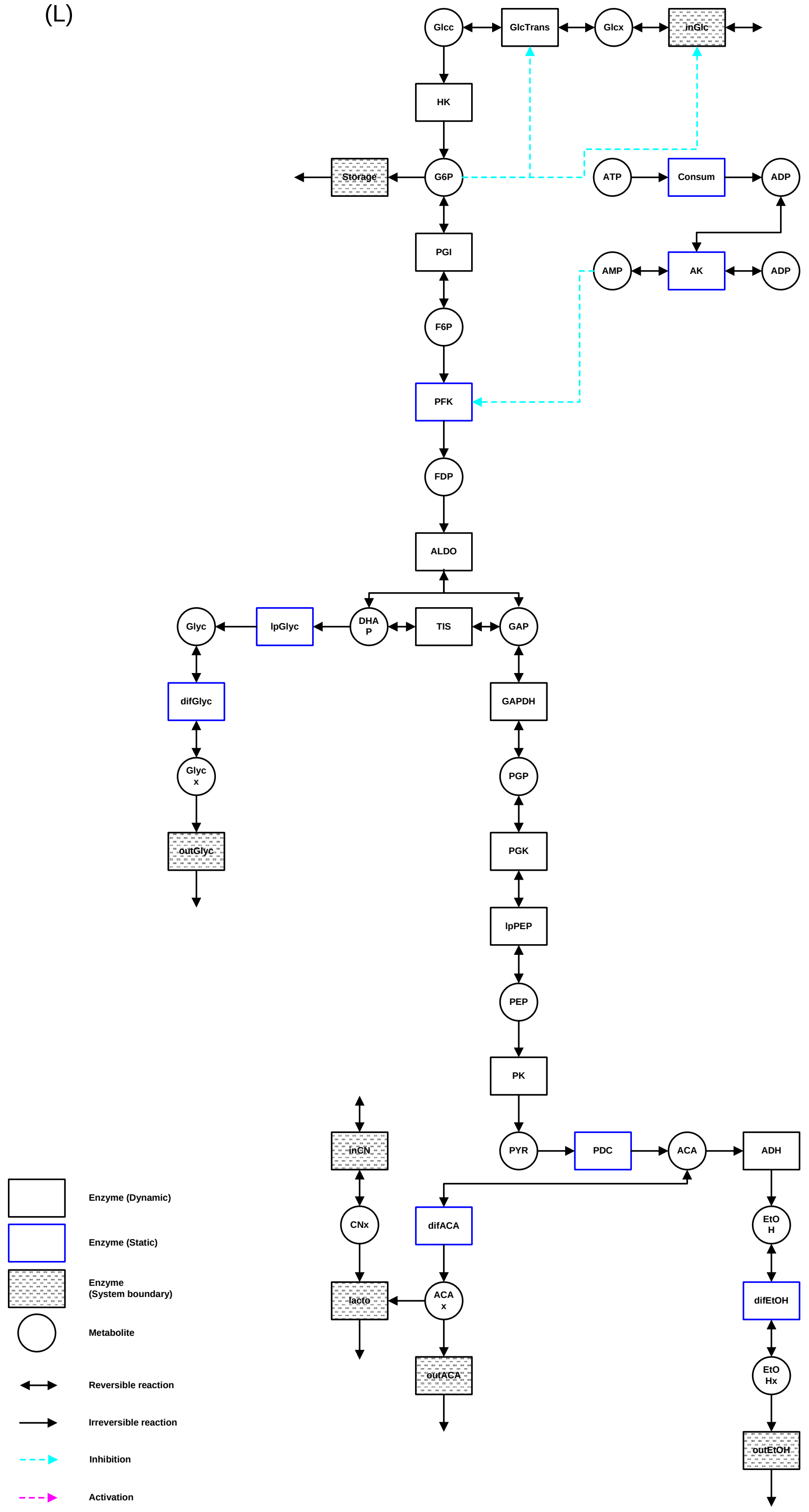

Figure S2 (Continued.)

(M)

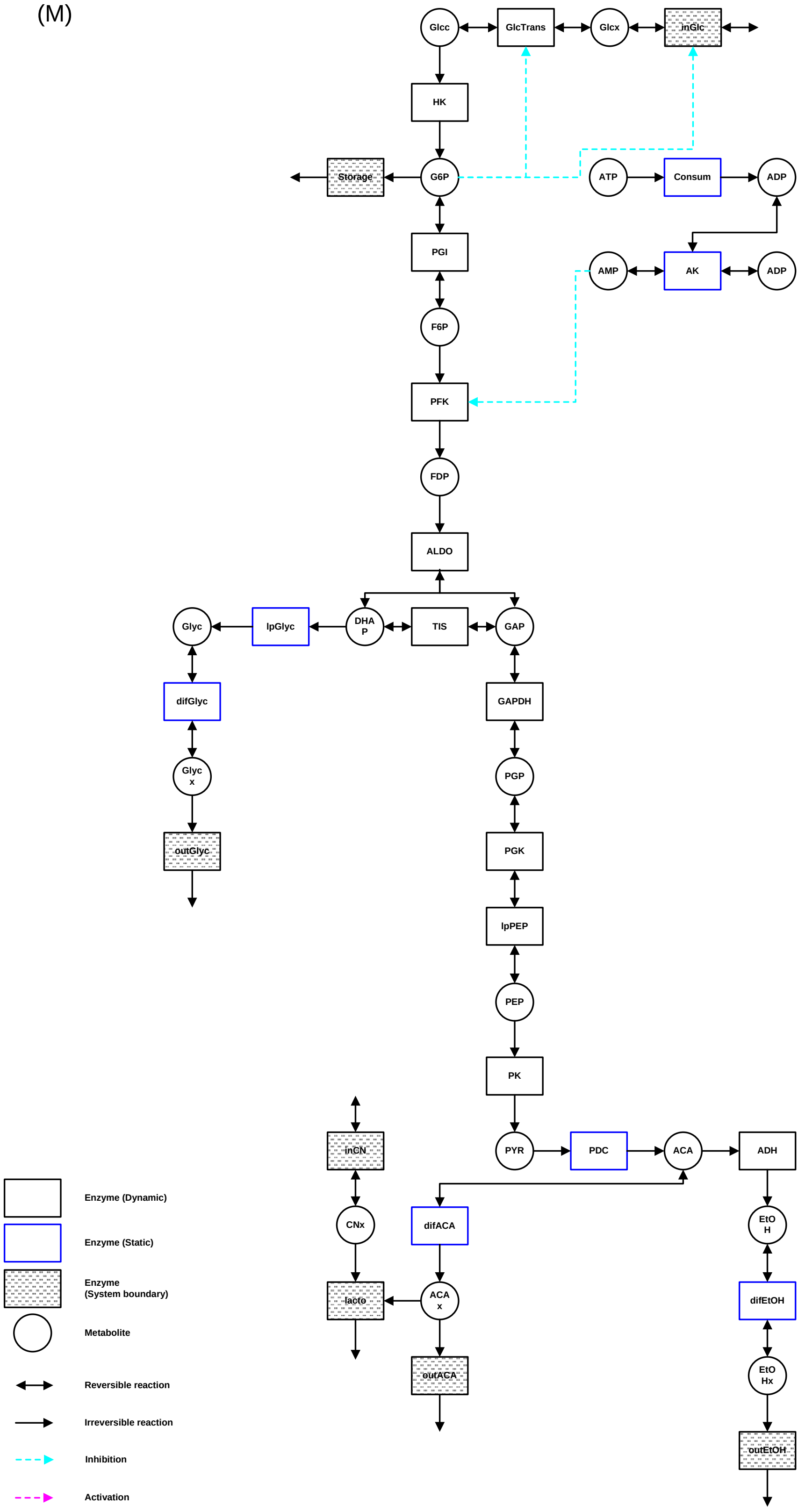

Figure S2 (Continued.)

(N)

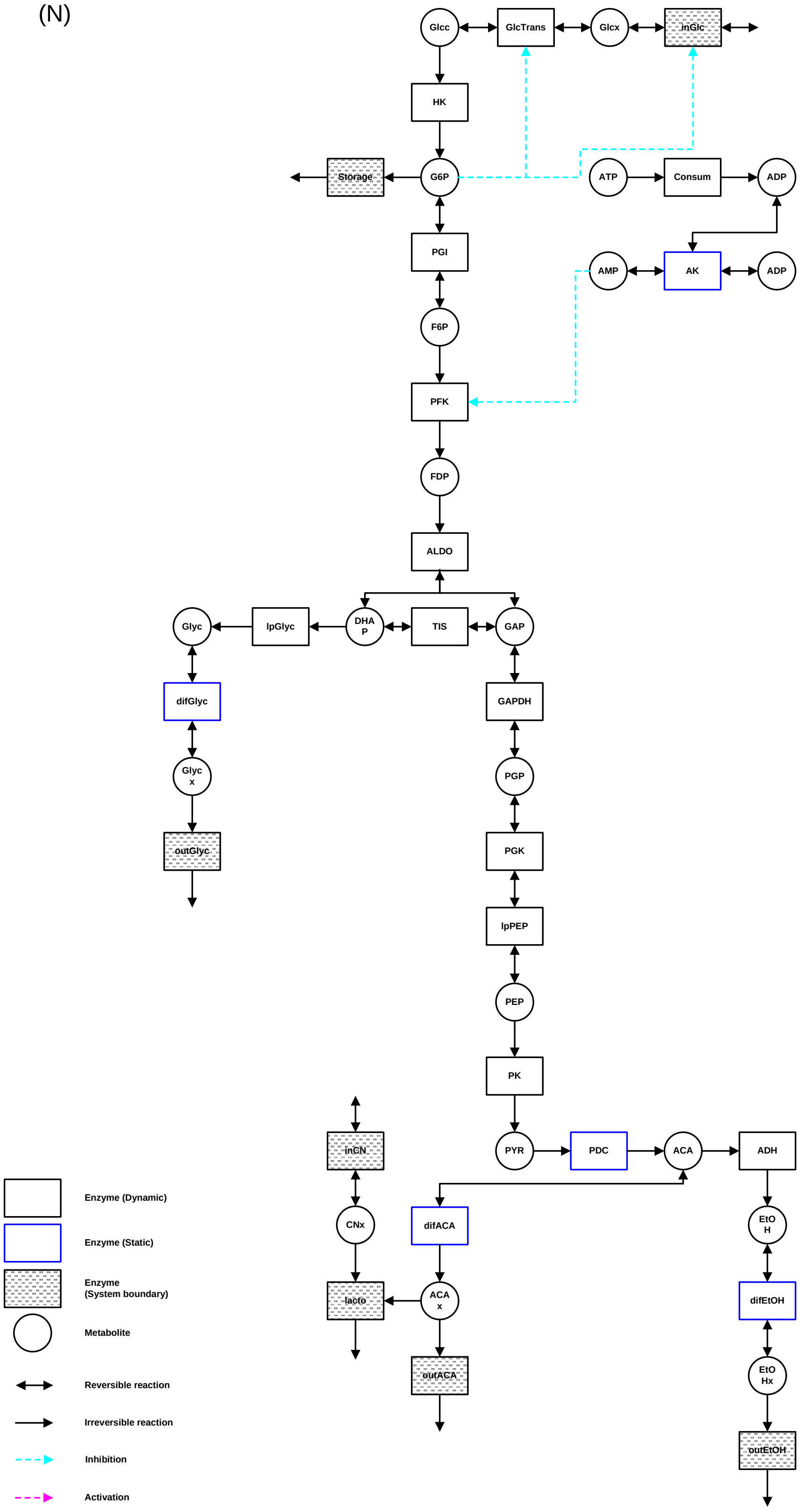

Figure S2 (Continued.)

(O)

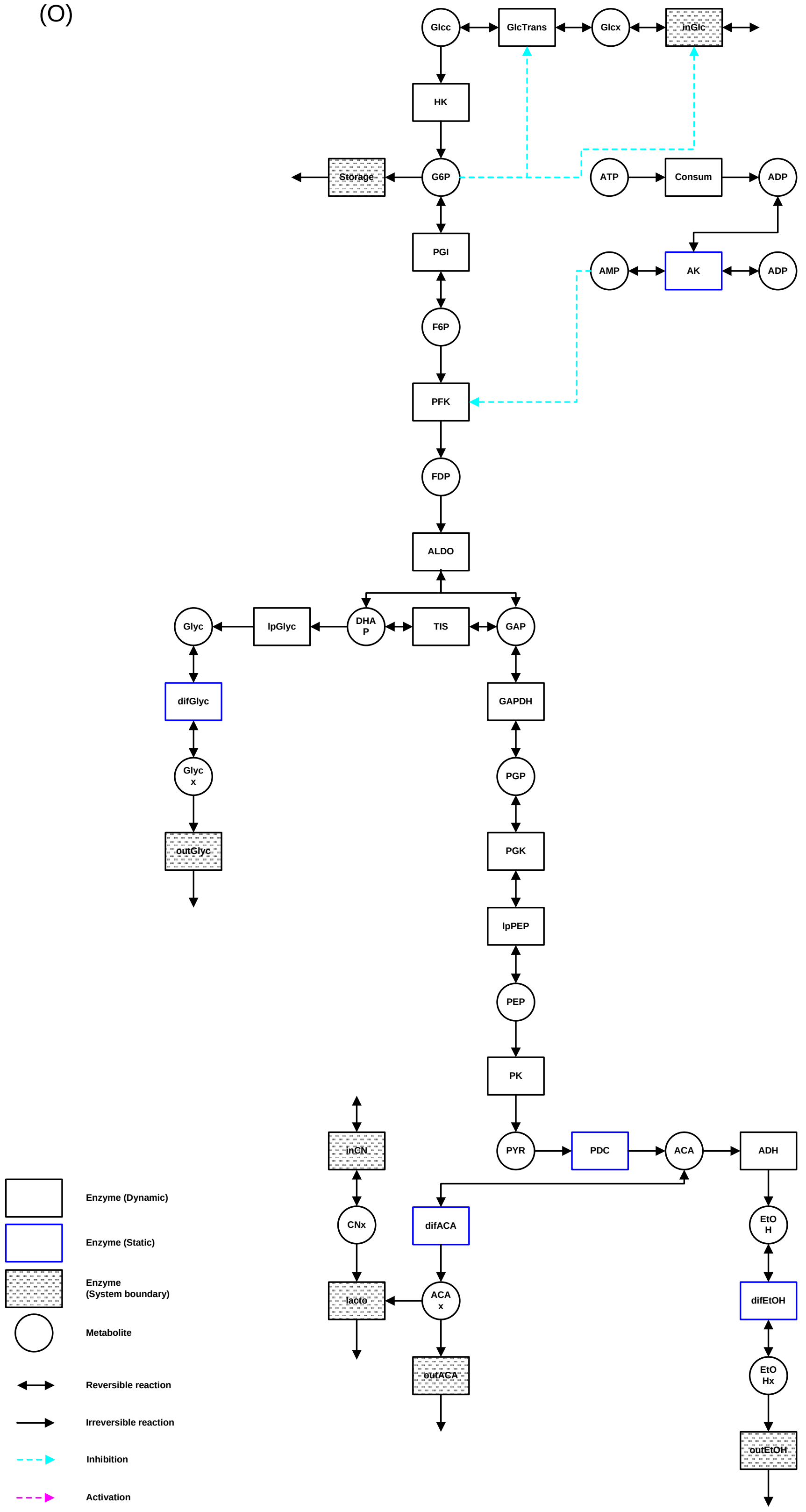

Figure S2 (Continued.)

(P)

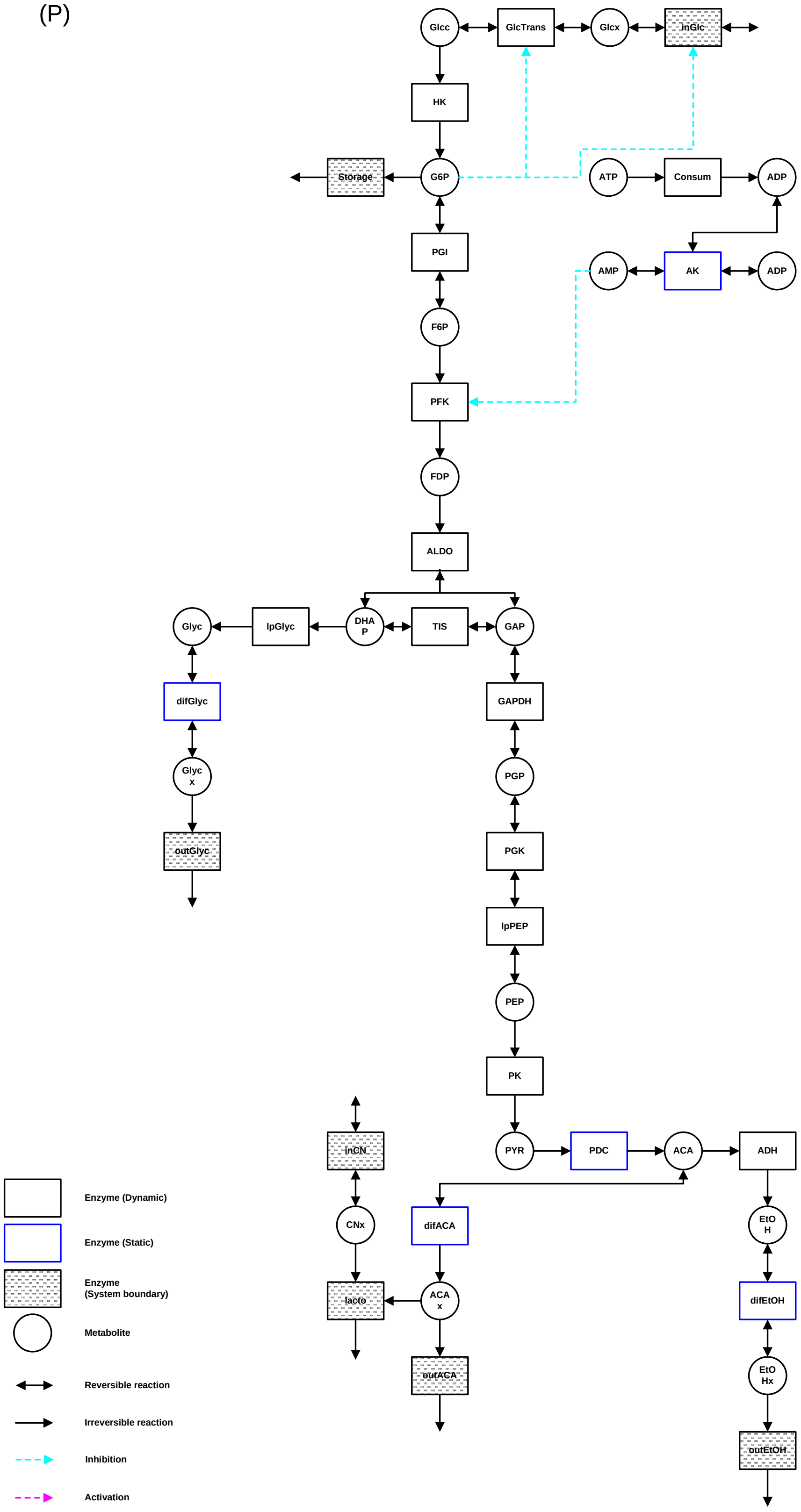

Figure S2 (Continued.)

(Q)

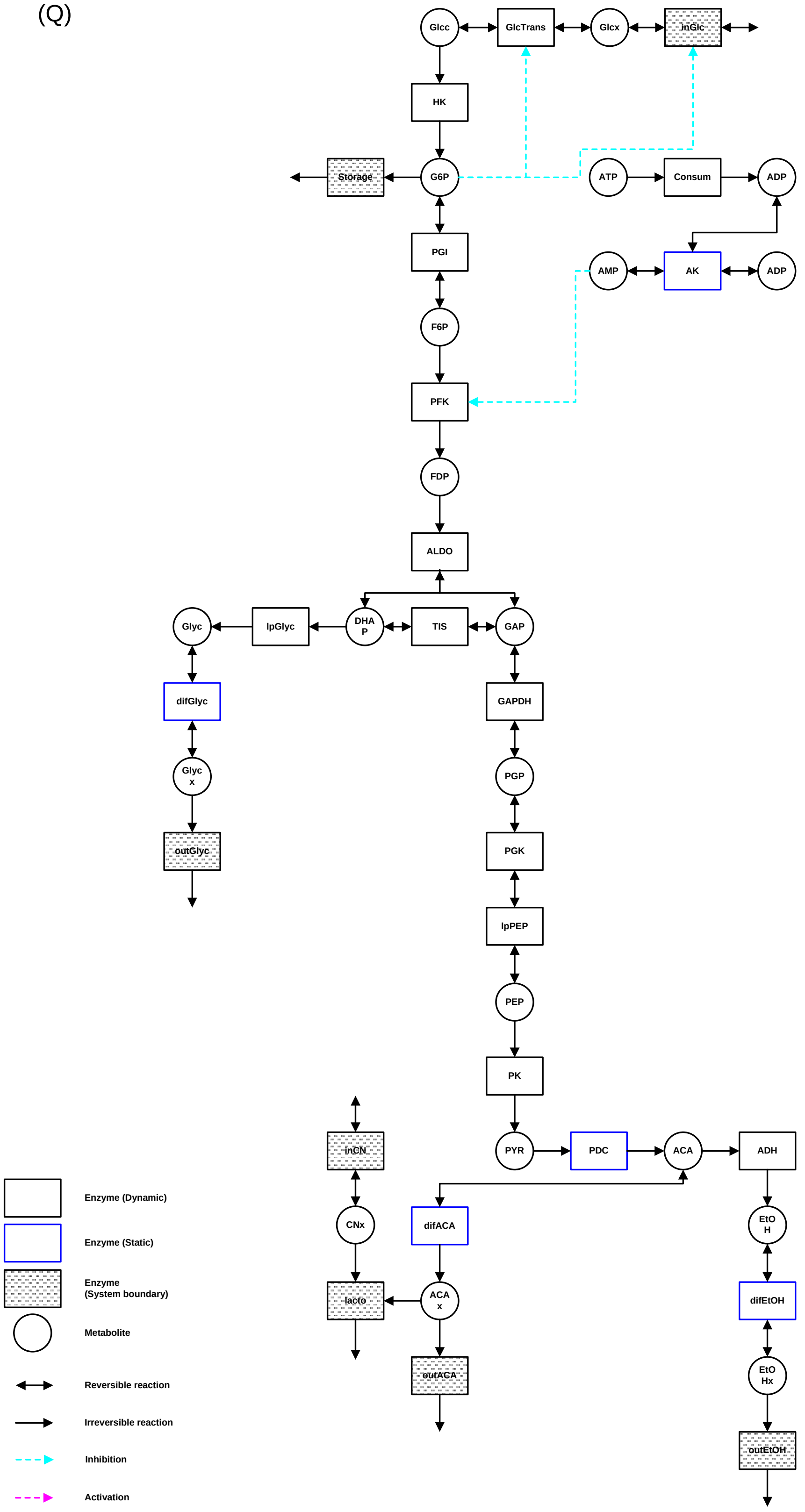

Figure S2 (Continued.)

(R)

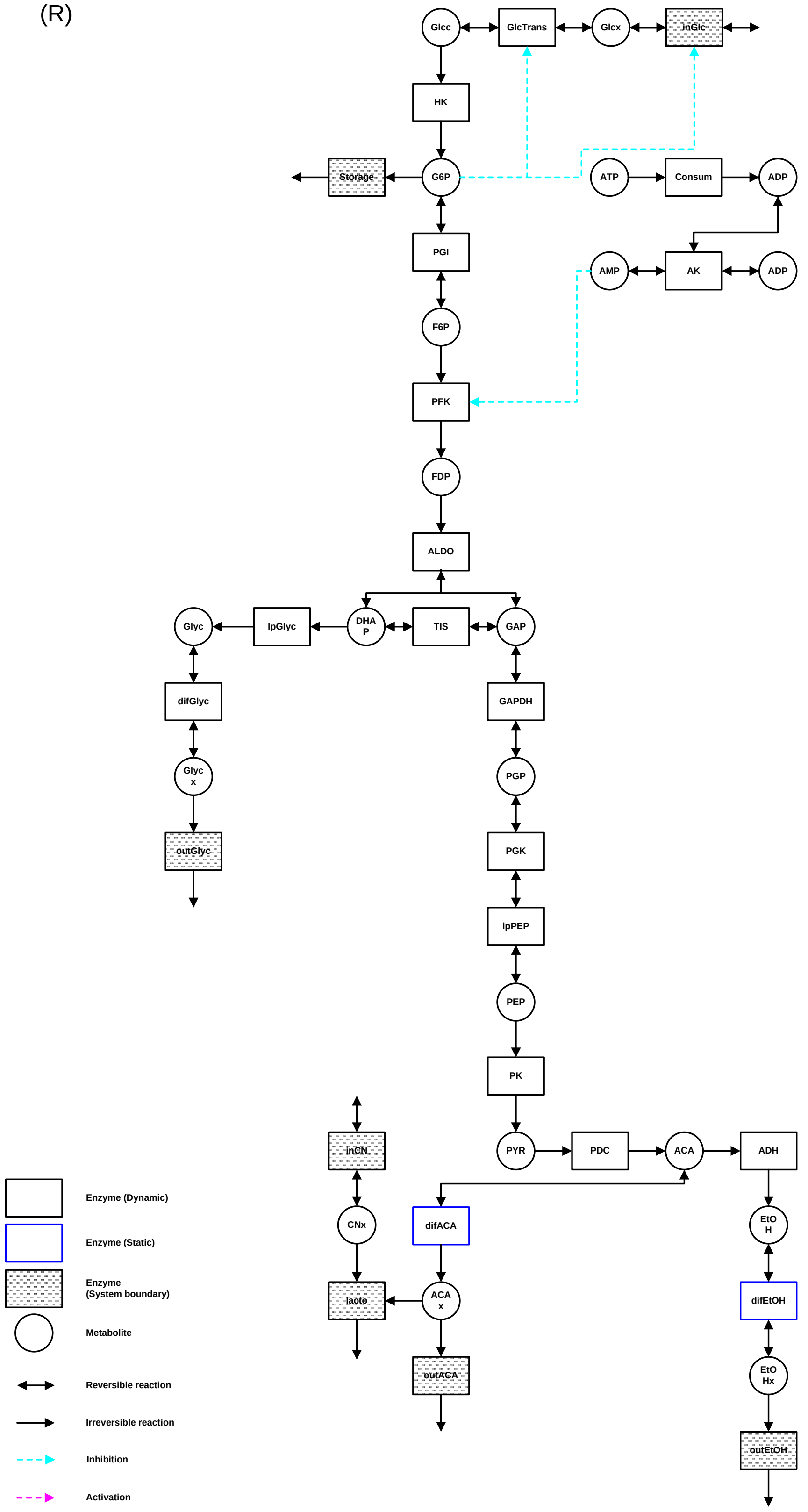

Figure S2 (Continued.)

Figure S2 Dynamic/static distinctions displayed on the metabolic map. (A) *E. coli*, weighting coefficient (*w*) = 1.000; (B) *E. coli*, *w* = 0.750; (C) *E. coli*, *w* = 0.500; (D) *E. coli*, *w* = 0.250; (E) *E. coli*, *w* = 0.100; (F) *E. coli*, *w* = 0.075; (G) *E. coli*, *w* = 0.050; (H) *E. coli*, *w* = 0.025; (I) *E. coli*, *w* = 0.010; (J) *S. cerevisiae*, *w* = 1.000; (K) *S. cerevisiae*, *w* = 0.750; (L) *S. cerevisiae*, *w* = 0.500; (M) *S. cerevisiae*, *w* = 0.250; (N) *S. cerevisiae*, *w* = 0.100; (O) *S. cerevisiae*, *w* = 0.075; (P) *S. cerevisiae*, *w* = 0.050; (Q) *S. cerevisiae*, *w* = 0.025; (R) *S. cerevisiae*, *w* = 0.010.

(A)

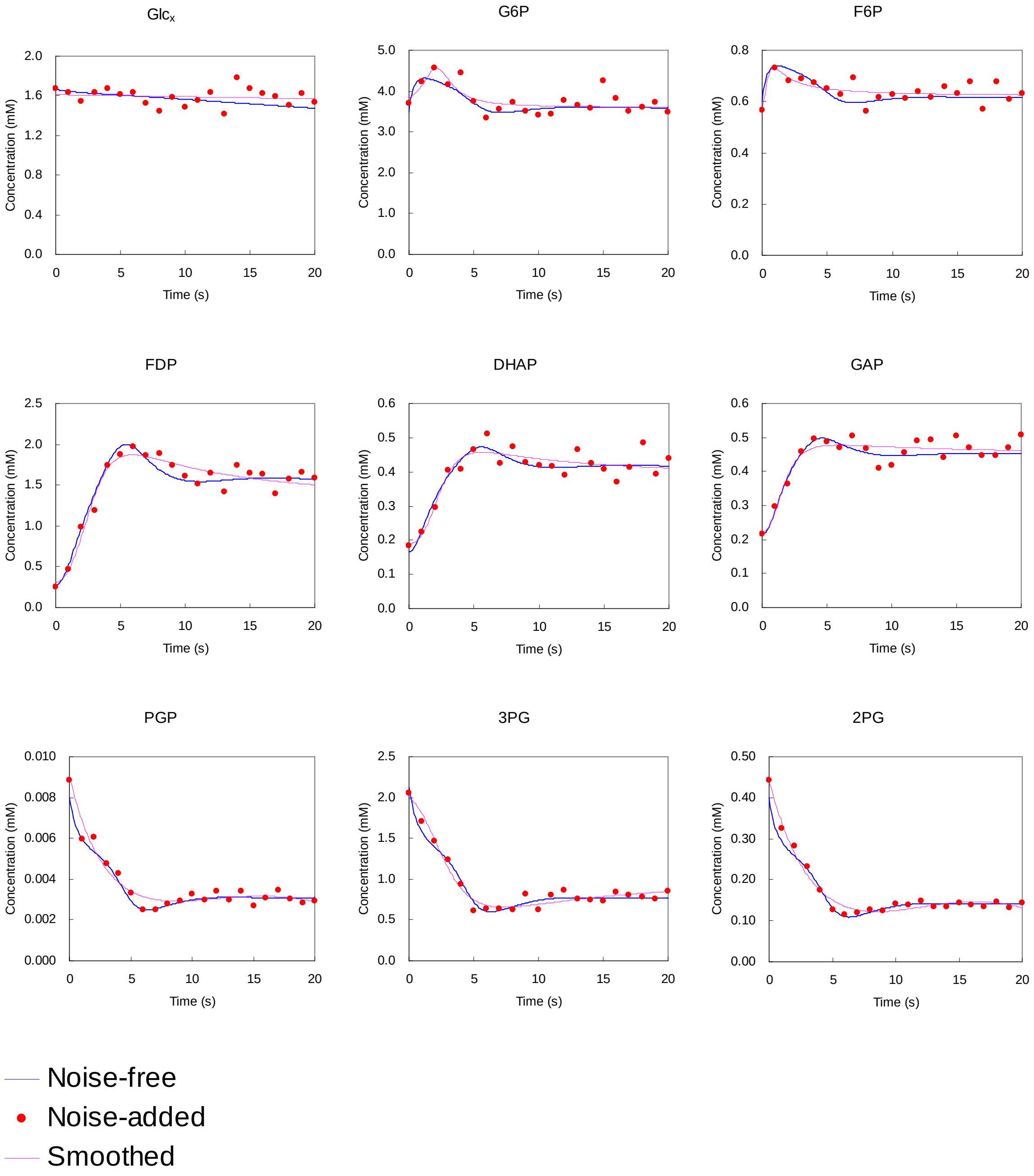
Glcx
G6P
F6P
FDP
DHAP
GAP
PGP
3PG
2PG
Concentration (mM)
Time (s)
Noise-free
Noise-added
Smoothed

Figure S3

(B)

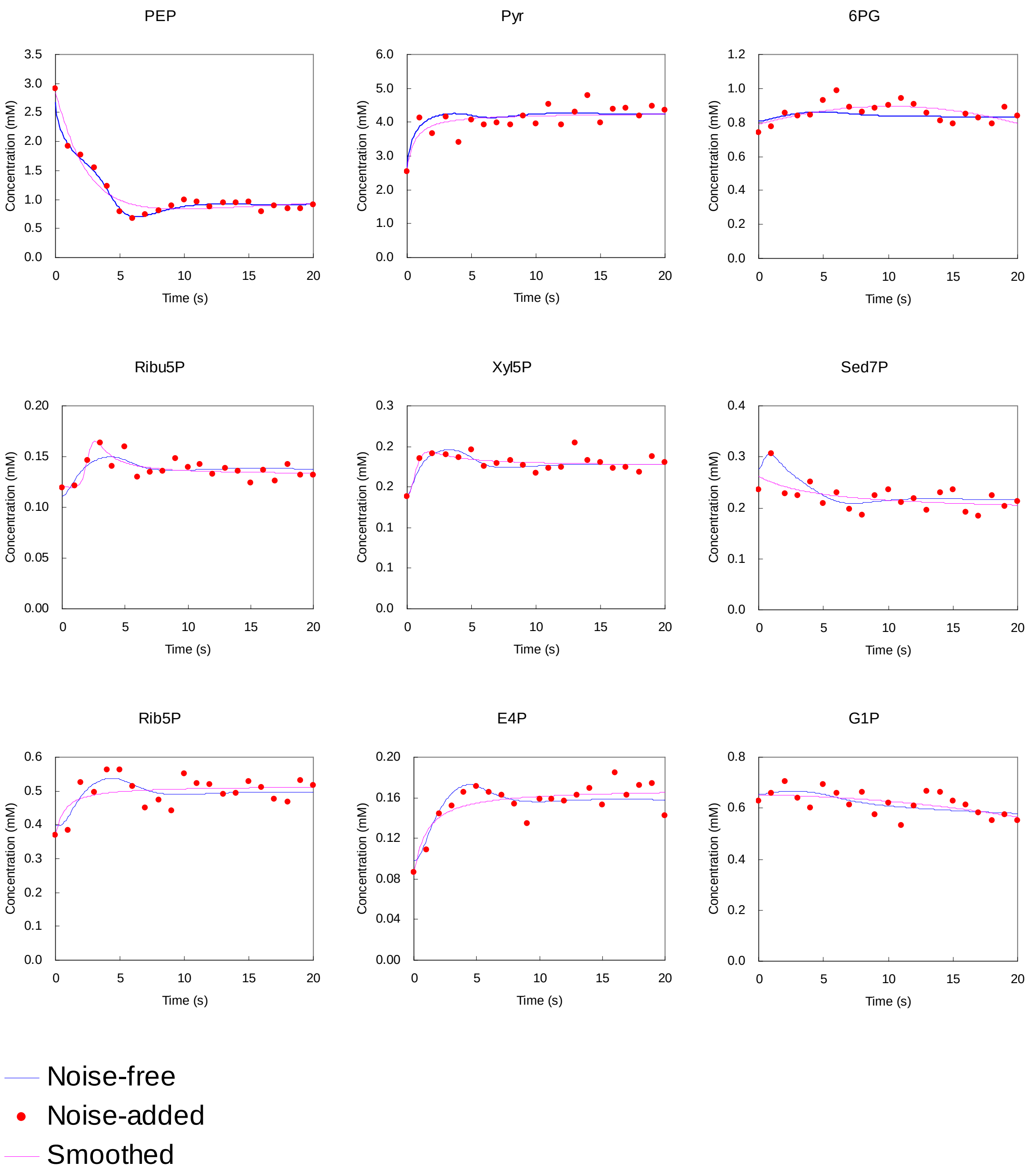

Figure S3 (Continued.)

(C)

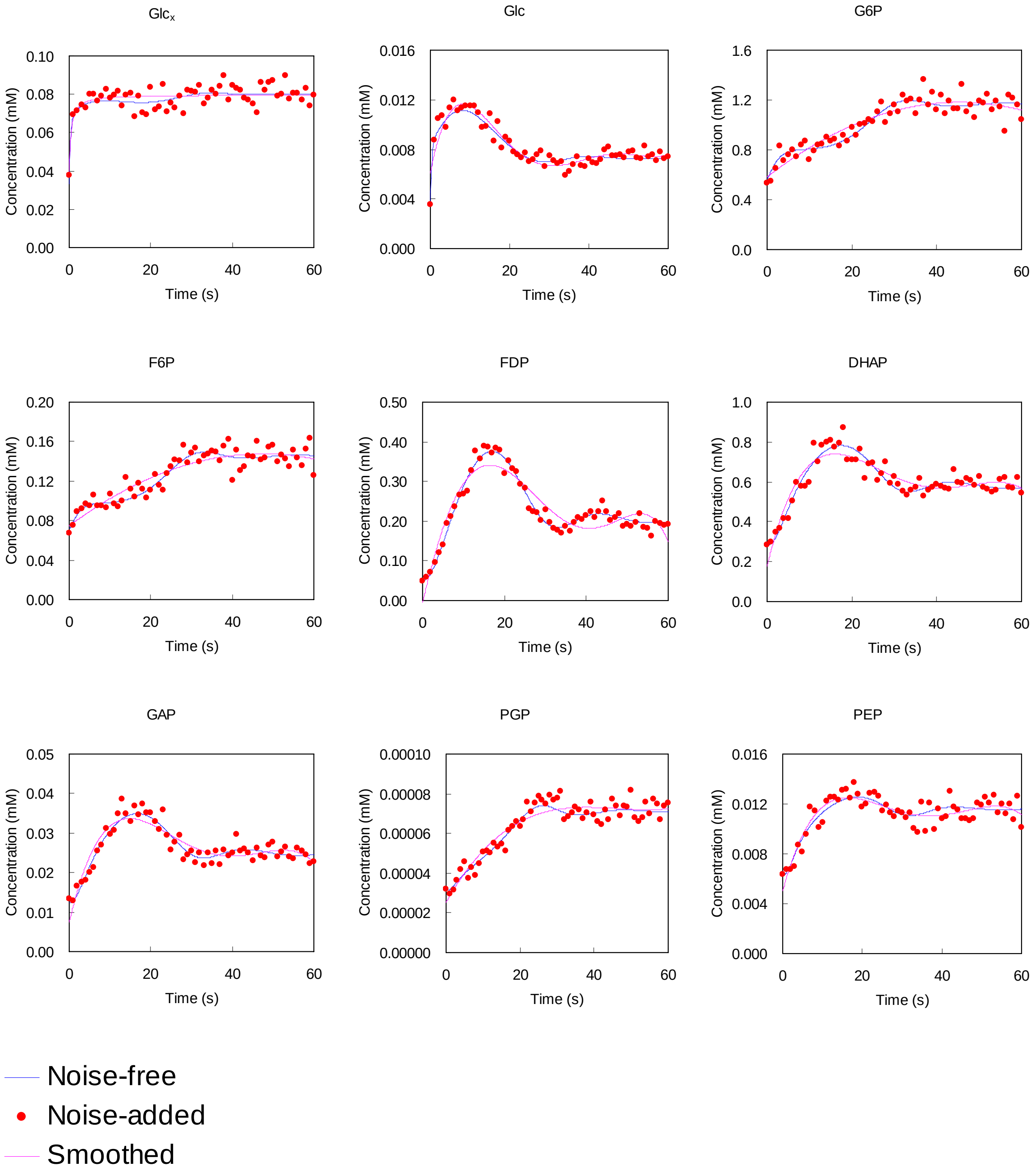

Figure S3 (Continued.)

(D)

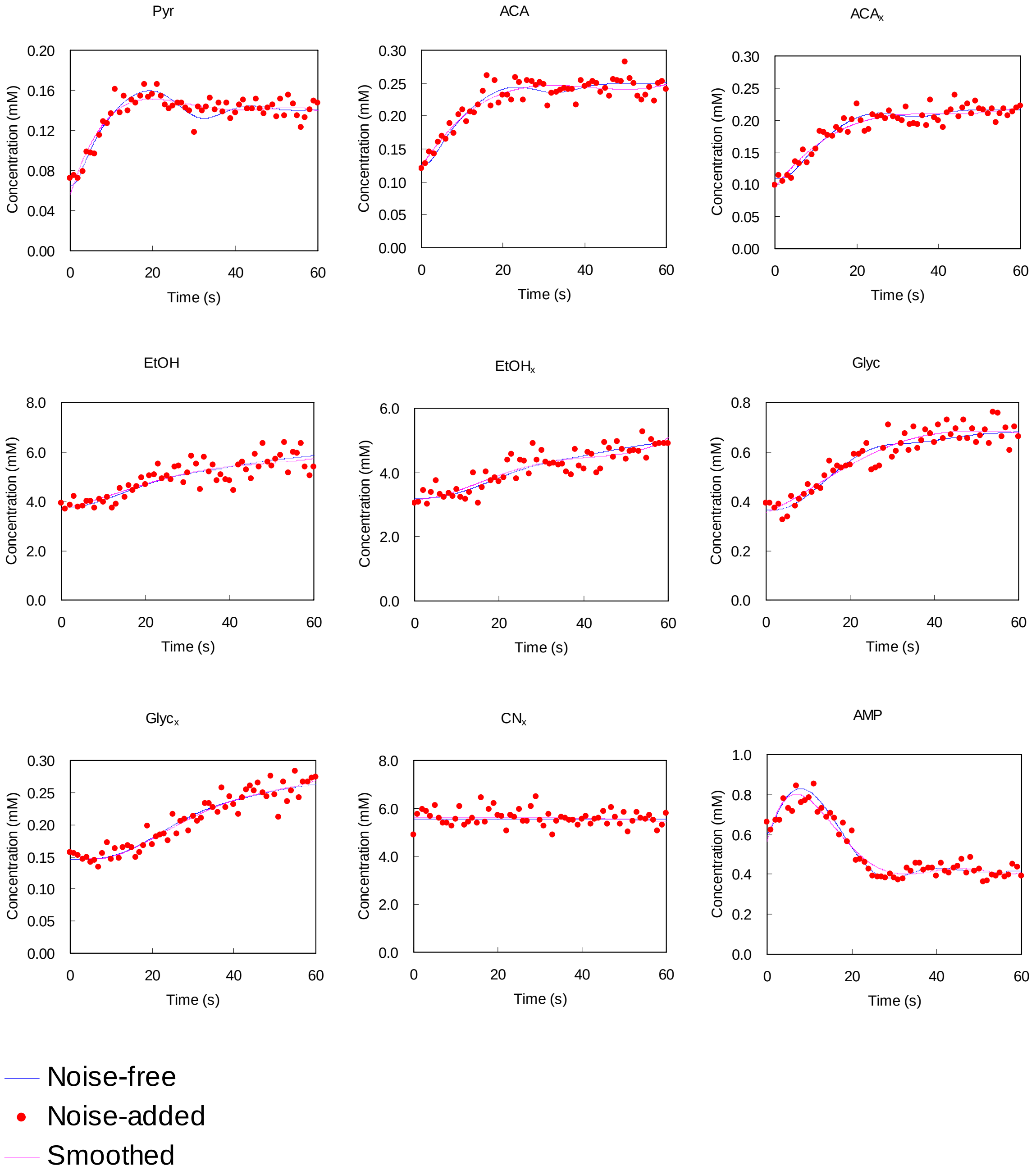

Figure S3 (Continued.)

(E)

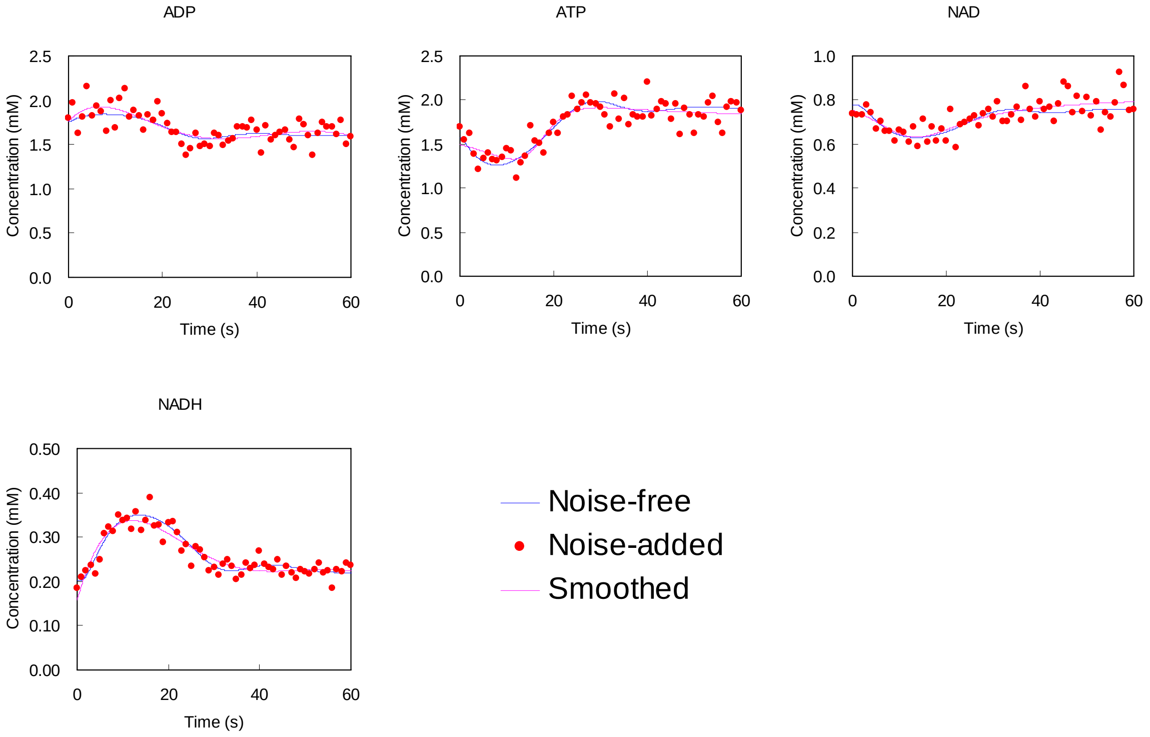

Figure S3 Noise-free, noise-added, and smoothed metabolite concentration time series. (A) and (B), *E. coli*; (C), (D), and (E), *S. cerevisiae*. Each noise-added data point is the average of five noise-added values.
